# Discovery of a novel class of benzimidazoles as highly effective agonists of bone morphogenetic protein (BMP) receptor signaling

**DOI:** 10.1038/s41598-022-16394-x

**Published:** 2022-07-15

**Authors:** Sheyda Najafi, Leonard Barasa, Sammy Y. Huang, Sabesan Yoganathan, Jeanette C. Perron

**Affiliations:** Department of Pharmaceutical Sciences, College of Pharmacy and Health Sciences, St. John’s University, Queens, NY USA

**Keywords:** Pharmacology, Receptor pharmacology, Biochemistry, Cell biology, Chemical biology

## Abstract

Increasing or restoring Bone Morphogenetic Protein receptor signaling is an effective therapy for conditions such as bone fracture and pulmonary arterial hypertension. However, direct use of recombinant BMPs has encountered significant obstacles. Moreover, synthetic, full agonists of BMP receptor signaling have yet to be identified. Here, we report the discovery of a novel class of indolyl-benzimidazoles, synthesized using a one-pot synthetic methodology, which appear to mimic the biochemical and functional activity of BMPs. The first-in-series compounds, SY-LB-35 and SY-LB-57, stimulated significant increases in cell number and cell viability in the C2C12 myoblast cell line. Cell cycle analysis revealed that these compounds induced a shift toward proliferative phases. SY-LB-35 and SY-LB-57 stimulated canonical Smad and non-canonical PI3K/Akt, ERK, p38 and JNK intracellular signaling pathways, similar to BMP2-stimulated responses. Importantly, increases in Smad phosphorylation and cell viability were dependent on type I BMP receptor activity. Thus, these compounds robustly activate intracellular signaling in a BMP receptor-dependent manner and may signify the first known, full agonists of BMP receptor signaling. Moreover, discovery of small molecule activators of BMP pathways, which can be efficiently formulated and targeted to diseased or damaged areas, could potentially substitute recombinant BMPs for treatment of BMP-related pathologies.

## Introduction

Bone Morphogenetic Proteins (BMPs) are a family of signaling factors that were initially discovered for the ability to regulate osteoblast lineage-specific differentiation of mesenchymal cells and later ectopic bone formation^1^. Now, these proteins are known to play crucial roles in many organ systems. BMPs are critical factors for embryogenesis and organ development, as well as for the maintenance of adult tissues^2–4^. Due to their widespread expression and importance as regulators throughout the body and because of their diverse functions in multiple organ systems, deficiencies in BMP production or functionality usually leads to marked defects or severe pathologies^2,3^.

Increasing or restoring BMP signaling has been shown to be therapuetically useful in reversing or reducing pulmonary arterial hypertension (PAH) progression^5,6^, in wound healing and the suppression of scar formation^7,8^, and in spinal fusion and fracture repair^9,10^. For various BMP-related health conditions, the direct use of recombinant BMPs (rBMPs) as therapeutic agents is the currently approved method, however, this approach faces significant obstacles. The requirement of very high concentrations of rBMP2 for bone fracture repair has created translational barriers due to high cost and complexity of formulation^11–13^. Thus, the discovery and development of small molecule agonists of BMP pathways, with therapeutic efficacy that can be efficiently formulated and delivered to target diseased areas, would be a substantial advance and could potentially replace the clincial use of rBMPs for the treatment of BMP-related disorders.

BMPs act as dimers and elicit their effects by binding to two types of serine-threonine kinase transmembrane receptors: type I and type II^14^. A pair of each type of BMP receptors are required for signal transduction and combine to form a tetrameric receptor complex. There are four type I BMP receptors (BMPRIA, BMPRIB, ALK1 and ALK2) and three type II BMP receptors (BMPR2, ActRIIA and ActRIIB), all of which can participate in forming heterotetrametric complexes to induce BMP signaling^15^. BMP signal transduction is classically mediated via the canonical Smad pathway of transcriptional regulators^16–18^. Multiple Smad-independent or non-canonical signaling pathways have also been identified downstream of BMP receptor activation. Some pathways, like the Smad pathway, regulate gene expression but other pathways elicit diverse effects on non-nuclear targets, such as the actin cytoskeleton^19–21^.

**Figure 1:**
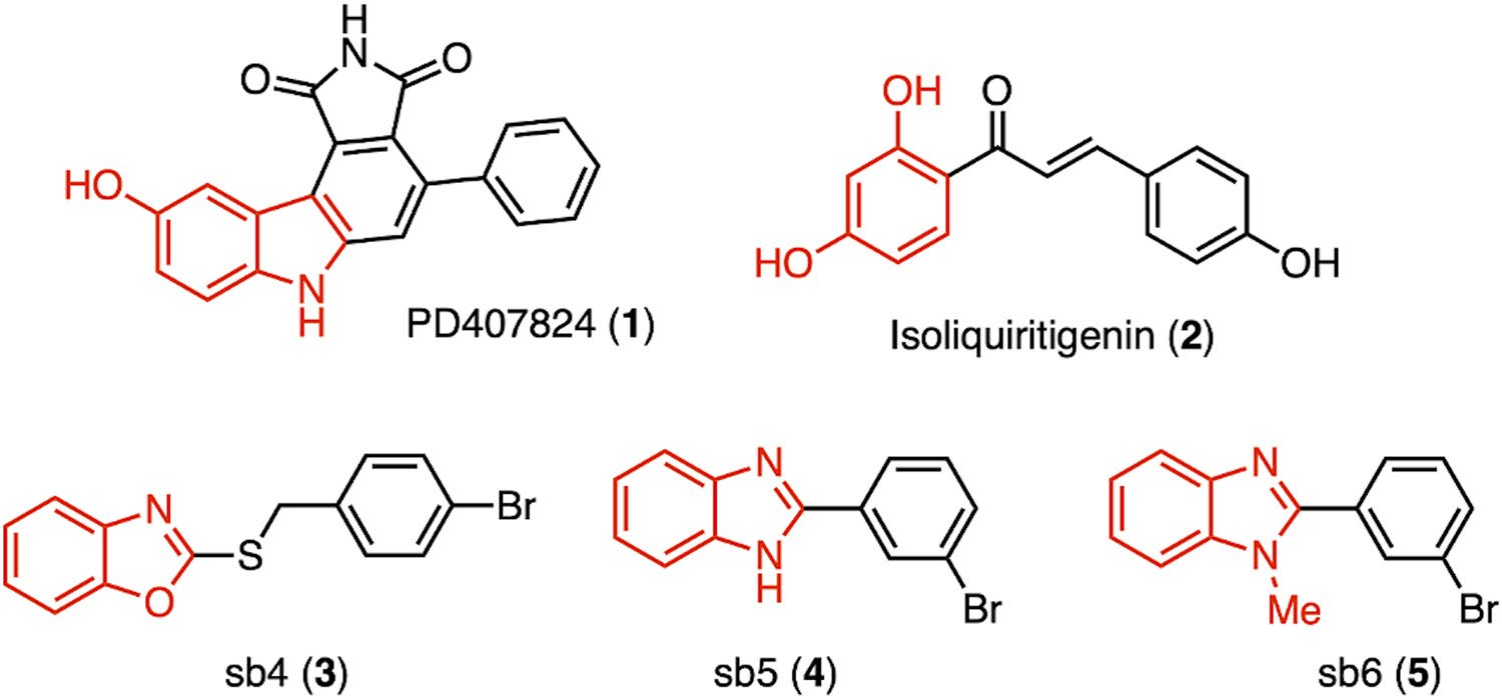
Reported small molecules that act as activators/partial agonists of BMP receptor-associated signaling. The figure illustrates the chemical structures of known BMP modulators. Images are modified from Feng et al*.* (compound **1**), Vrijens et al*.* (compound **2**) and Bradford et al. (compounds **3**, **4** and **5**). Compounds **1**, **3**, **4**, and **5** are synthetic molecules, while compound **2** is a natural product^13,22,23^.

In an attempt to synthesize an effective small molecule agonist of BMP signaling pathways, several small molecules that either enhance or synergistically improve BMP activity were considered in our synthetic design for new classes of molecules (Fig. 1)^13,22–24^. PD407824 (**1**), a carbazole derivative, was reported as a sensitizer of subthreshold levels of BMP4 in C2C12 myoblast cells and human embryonic stem cells. However, this sensitization appears to be the result of an indirect mechanism independent of BMP receptors^22^. Benzoxazoles and benzimidazoles (compounds **3–5**) are classes of small molecules identified by high-throughput screening (HTS) using a luciferease reporter^13^. Compound **3** (a benzoxazole), in particular, increased the phosphorylated forms of Smad1/5/8 (p-Smads) and led to the activation of BMP target genes, such as Id1 and Id3^13^. This compound (**3**, sb4) appears to allosterically activate the type I BMP receptor as Smad-dependent signaling was observed even in the presence of an extracellular inhibitor of BMP signaling or a type I BMP receptor inhibitor. Compounds **4** and** 5** are benzimidazole class molecules and were shown to enhnace BMP signaling as well^13^. Finally, Roussel and coworkers reported two unique classes of small molecules as potential activators of BMP signaling^23,24^. Once again, a HTS led to the identification of two natural chalcones (isoliquiritigenin and 4’hydroxychalcone) and three synthetic compounds that target BMP signaling. These compounds induced the phosphorylation of Smad1/5/8 in a dose dependent manner^23,24^.

**Figure 2:**
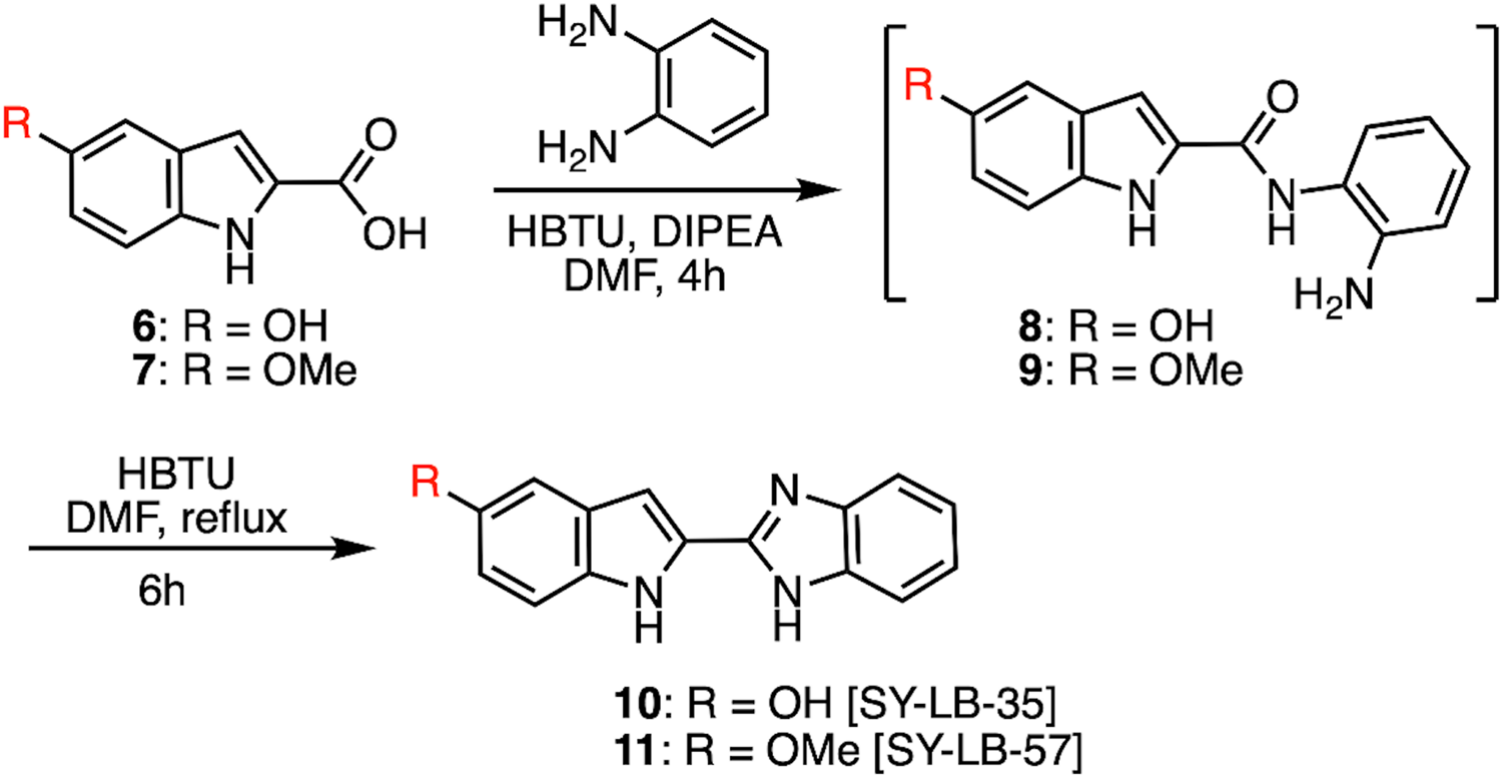
Synthesis and structures of indolyl-benzimidazoles. The figure describes the synthetic scheme used to prepare the two indolyl-benzimidazoles. The carboxylic acid (compounds **6** and **7**) was converted after 4 h to the amide (compounds **8** and **9**) in the first step, and the amide was subsequently converted after 6 h into the benzimidazole (compounds **10** and **11**) via a one-pot process.

Careful assessment of these previously reported small molecules identified specific heterocyclic scaffolds, such as indole, benzoxazole and benzimidazole, that may be characterized as useful chemical space to explore. Indeed, the Yoganathan lab has been investigating the biochemistry and biology of structurally diverse benzimidazoles and established a library of small molecules^25,26^. Based on the heterocylic scaffolds in such BMP signaling modulators as compounds **1**, **3**,** 4** and **5** (Fig. 1), two distinct indolyl-benzimidazoles (compounds **10** and **11**; Fig. 2) were designed and synthesized as structurally novel, small molecules for our initial studies. Moreover, the Yoganathan lab is poised to generate a larger library of similar small molecules for further exploration of the structure–activity relationships of indolyl-benzimidazole derivatives.

Herein, this study presents the synthetic approach and initial pharmacological evaluation of two new indolyl-benzimidazole compounds (SY-LB-35 and SY-LB-57). The one-pot synthesis^27^ combined with the low cost and stability of these compounds is an advance over other benzimidazole synthesis methods. The novel compounds showed signs of toxicity only at high mircomolar concentrations. SY-LB-35 and SY-LB-57 stimulated large increases in cell viability, which is likely due to stimulation of proliferation, given that these compounds also increased cell number with respect to control and caused shifts towards the S and G2/M phases of the cell cycle. These compounds stimulated robust increases in phospho(p)-Smad1/5/8 (p-Smad), p-Akt, p-ERK and p-p38 levels. Moreover, inhibition of type I BMP receptor activity blocked increases in p-Smad and cell viability induced by SY-LB-35 and SY-LB 57. Taken together, these data suggest that the novel indolyl-benzimidazoles, SY-LB-35 and SY-LB-57, are the first reported small molecules with true BMP receptor agonist activity.

## Results

### Development of a new one-pot synthesis procedure

The two indolyl-benzimidazoles (SY-LB-35 and SY-LB-57) were prepared using a highly efficient synthetic approach as described in Fig. 2^27^. The first step in the synthesis led to the conversion of indole-2-carboyxlic acid to the corresponding aryl amide. The use of DMF as solvent provided the needed solubility and the high boiling point of DMF allowed us to perform the reflux in the second step of the synthesis. The formation of amide was confirmed by TLC and LC/MS analysis. Without isolating the amide, the synthesis continued with the conversion of the amide to the corresponding benzimidazole using an HBTU-promoted protocol. The final compounds were obtained in high yield (78%–92%) after a two-step purification sequence, which included a silica gel column chromatography and recrystallization. Both compounds were fully characterized using NMR spectroscopy and mass spectrometry (see Supplemental Materials and Supplemental Figs. S1 and S2).

### SY-LB-35 and SY-LB-57 significantly increase the cell viability of C2C2 cells

To first determine how these novel compounds effect the health of C2C12 cells and to establish the range of subtoxic concentrations for future studies, cell viability assays were carried out. Serum-starved C2C12 cells were exposed to increasing concentrations (0.01–1000 µM) of SY-LB-35 and SY-LB-57 for 24 h. Exposure to Triton X-100 (125 µM) is toxic to cells and was used as a negative control for cell viability. Following the 24-h exposure period, the MT Cell viability substrate was added to the cultures. Only viable cells can reduce the substrate and, thereby, generate a luminescent signal. Any decrease in luminescence was considered an indication of cell death or toxicity. In contrast, increases in luminescence signals are typically due to proliferation of cells in the treated cultures.

**Figure 3:**
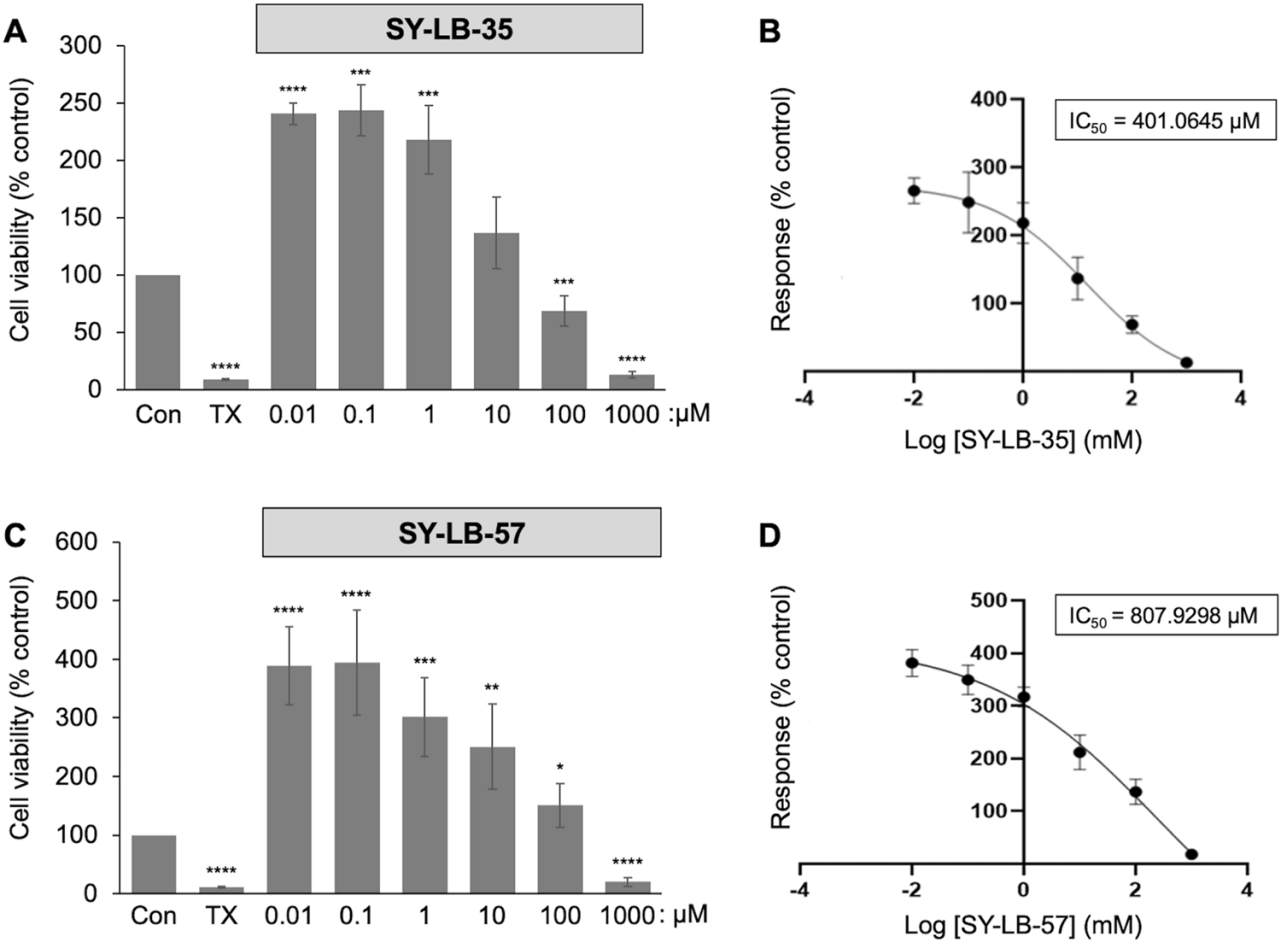
Significant increases in C2C12 cell viability are stimulated by SY-LB-35 or SY-LB-57. C2C12 cells were serum-starved overnight and treated with the indicated concentrations of SY-LB-35 (**A**) or SY-LB-57 (**C**) for 24 h. Treatments with Triton X-100 (TX, 125 µM) was used as negative control for viability in the MT Glo assay. Cell viability is presented as a percent of luminescence detected in control, untreated cells. (**A**) Treatment of C2C12 cells with SY-LB-35 at 100 µM and 1 mM significantly reduced cell viability when compared to control cells (69%, ***p < 0.001 and 13%, ****p < 0.0005%; respectively). In contrast, at lower concentrations of 0.01 µM, 0.1 µM and 1 µM, the cell viability was significantly increased (241%, ****p < 0.0005; 244%, and 218%, ***p < 0.001, respectively). Cell viability measured at 10 µM SY-LB-35 was not different from control (137%). Data are expressed as mean ± SEM (n = 3) with each experiment performed in triplicate. (**B**) Non-linear regression analysis reveals an IC_50_ value of 401.0645 µM for SY-LB-35. (**C**) Treatment of C2C12 cells with SY-LB-57 at 1 mM reduced cell viability significantly (20%, ****p < 0.0005). Significant increases in cell viability were observed in response to all other concentrations tested (0.01 µM: 390%; 0.1 µM: 394%, ****p < 0.0005; 1 µM: 301%, ***p < 0.001; 10 µM: 251%, **p < 0.01; 100 µM: 151%, *p < 0.05). Data are expressed as mean ± SEM (n = 3) with each experiment performed in triplicate. (**D**) Non-linear regression analysis reveals an IC_50_ value of 807.9298 µM for SY-LB-57.

In response to treatment of C2C12 cells with SY-LB-35, the highest concentrations (100 µM and 1 mM) significantly decreased cell viability compared with control (30% decrease; ***p < 0.001 and 80% decrease; ****p < 0.0005, respectively; Fig. 3A). At lower concentrations (0.01 µM to 10 µM), no decreases in cell viability were observed indicating that SY-LB-35 was well tolerated by C2C12 cells. Instead, statistically significant increases in cell viability were observed following treatment with concentrations less than 100 µM compared to control (0.01 µM, 150% over control; ****p < 0.0005; 0.1 µM and 1 µM, 150% and 120% over control, respectively; ***p < 0.001; Fig. 3A). A non-linear curve analysis was carried out to calculate the IC_50_ value allowing determination of the concentration of SY-LB-35 at which the cell viability is reduced by 50%. The IC_50_ value for SY-LB-35 in C2C12 cells was 401.06 µM (Fig. 3B). These results indicate that concentrations of SY-LB-35 from 0.01 to 10 µM are non-toxic and can be used for future experiments.

Treatment with 1 mM SY-LB-57 also caused a highly significant decrease in cell viability in C2C12 cultures compared with control (85% decrease; ****p < 0.0005; Fig. 3C). In contrast, concentrations of 0.01–100 µM SY-LB-57 increased cell viability in C2C12 cells significantly compared with control (0.01 µM and 0.1 µM, 280% and 290% over control, respectively, ****p < 0.0005; 1 µM, 200% over control; ***p < 0.001; 10 µM, 150% over control, **p < 0.01; 100 µM, 50% over control, *p < 0.05; Fig. 3C). Non-linear analysis demonstrates an IC_50_ value of 807.93 µM for SY-LB-57 (Fig. 3D). Increases in cell viability were induced by BMP2, BMP9, BMP6 and BMP7, which are members of three different BMP subfamilies indicating that increases in cell viability is an activity shared among BMP family members (Supplemental Fig. S3). SY-LB-35 demonstrated nearly identical activity to that exhibited by four different rBMPs In contrast, the extent of cell viability induction by SY-LB-57 was 200% greater than either SY-LB-35- or BMP-induced responses.

**Figure 4:**
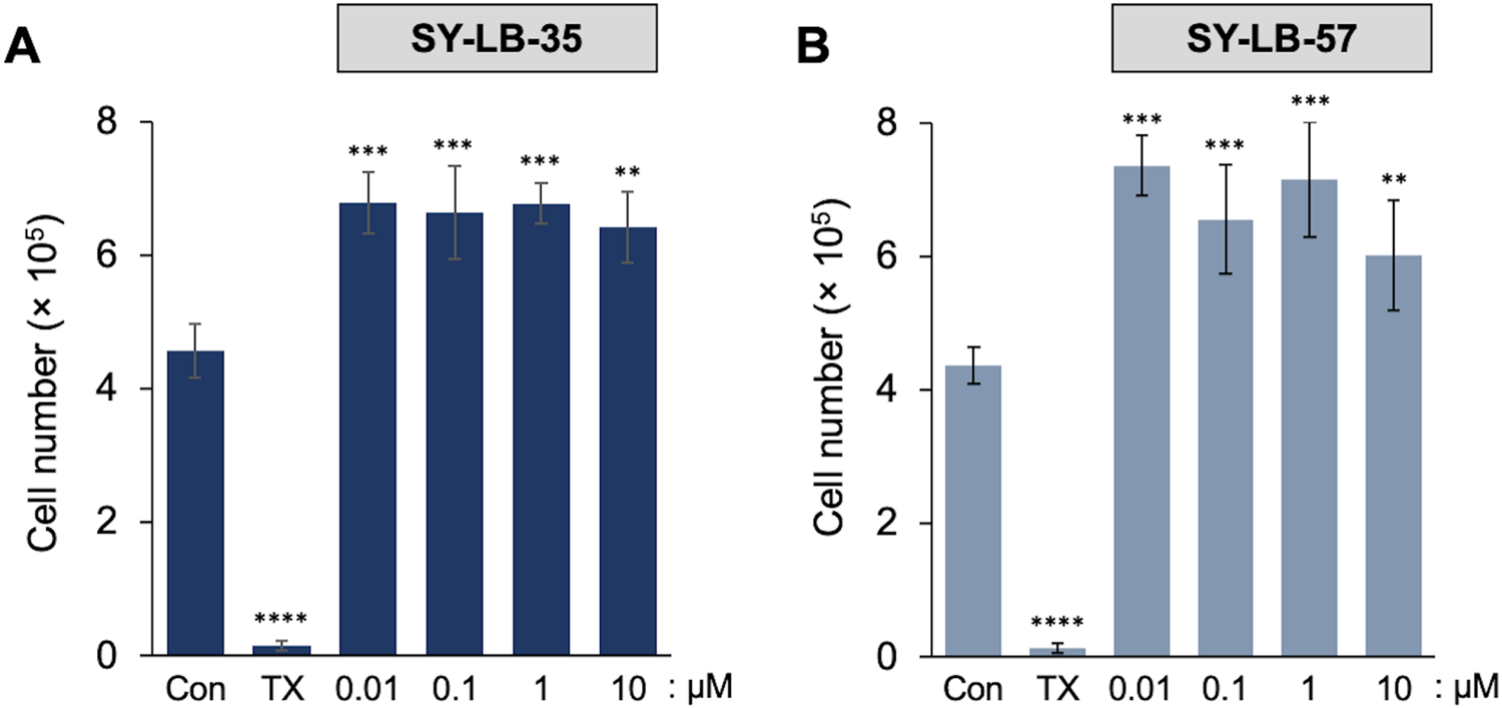
SY-LB-35 and SY-LB-57 increased cell concentration in C2C12 cells. Serum-starved C2C12 cells were treated with (**A**) SY-LB-35 and (**B**) SY-LB-57 (0.01–10 µM) for 24 h. Triton X-100 (TX) was used as negative control. C2C12 cells were collected after trypsinization, and the cell count was measured using Propidium iodide/Acridine orange. (**A**) Treatment with SY-LB-35 caused a significant increase in cell number compared with control, untreated cells (Con: 4.6 × 10^5^ cells; 0.01 µM: 6.8 × 10^5^ cells, 0.1 µM: 6.6 × 10^5^ cells, 1 µM: 6.8 × 10^5^ cells, ***p < 0.001; 10 µM: 6.4 × 10^5^ cells, **p < 0.01). (**B**) Treatment with SY-LB-57 caused a significant increase in cell number compared with control, untreated cells (Con: 4.4 × 10^5^ cells; 0.01 µM: 7.4 × 10^5^ cells, 0.1 µM: 6.5 × 10^5^ cells, 1 µM: 7.2 × 10^5^ cells, ***p < 0.001; 10 µM: 6.0 × 10^5^ cells, **p < 0.01). Data are expressed as mean ± SEM (n = 3) and each experiment was conducted in triplicate.

To further investigate how SY-LB-35 and SY-LB-57 cause increases in cell viability, the total cell number was determined following a 24-h treatment of C2C12 cells with 0.01–10 µM SY-LB-35 or SY-LB-57. The live cell counts demonstrated that SY-LB-35 and SY-LB-57 increased cell numbers significantly compared with cultures grown in Serum-Starvation (SS) medium alone (Fig. 4, ***p < 0.001; **p < 0.01; *p < 0.05). For the concentrations tested, SY-LB-35 increased the number of cells by an average of approximately 40% (Fig. 4A) and SY-LB-57 enhanced cell numbers to an average increase of 70% compared to the control (Fig. 4B). Taken together, these data demonstrate that SY-LB-35 and SY-LB-57 are safe to use at concentrations at 10 µM and less. Moreover, both novel compounds induce a substantial proliferative effect in C2C12 cells.

### SY-LB-35 and SY-LB-57 strongly stimulate Smad phosphorylation and nuclear translocation

**Figure 5:**
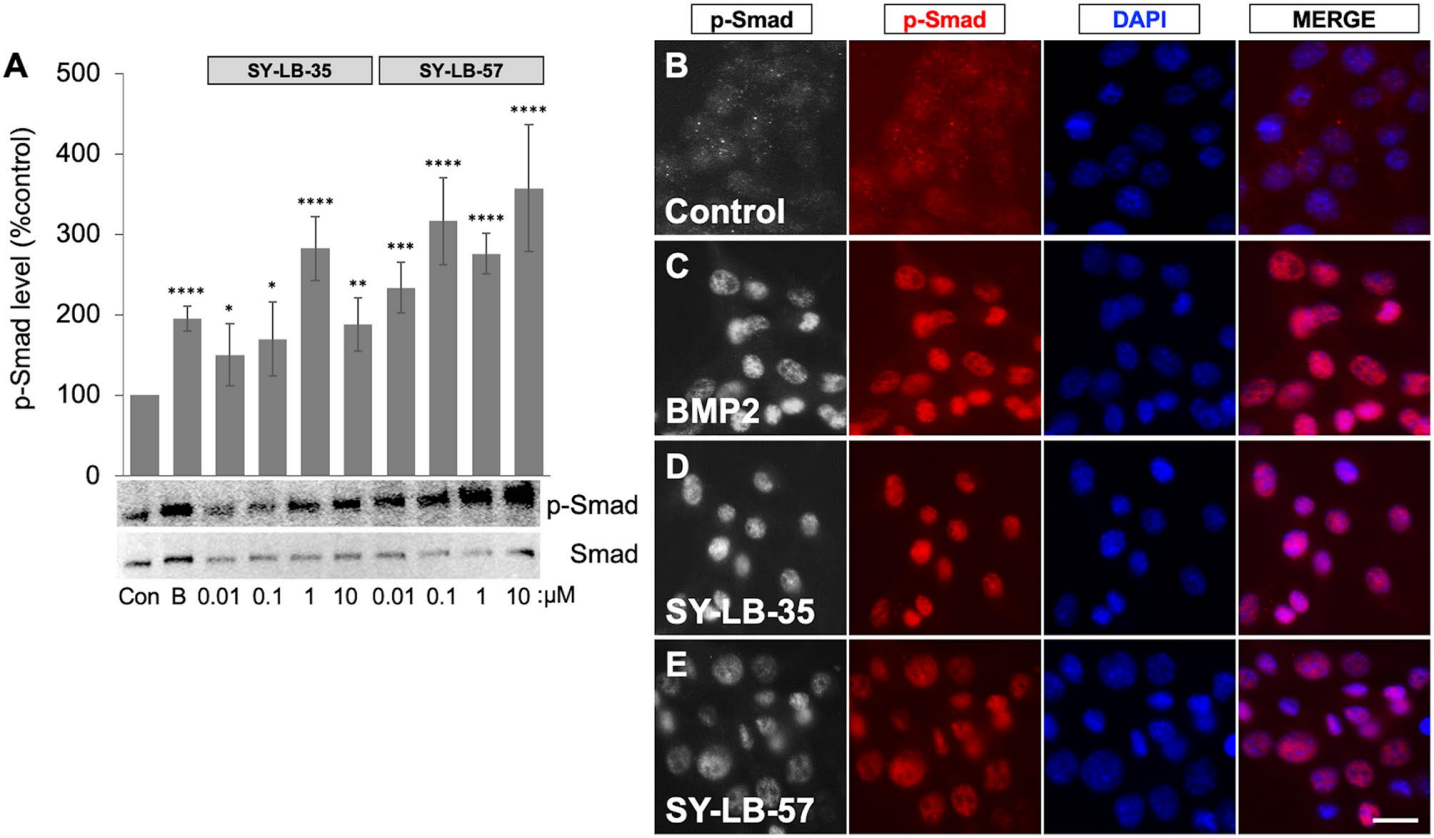
Smad phosphorylation and nuclear translocation in the presence of SY-LB-35 or SY-LB-57. (**A**) Serum-starved C2C12 cells were treated with increasing concentrations of SY-LB-35 or SY-LB-57 (0.01–10 µM) for 30 min. BMP2 (50 ng/mL) was used as a positive control. Western blot analysis using anti-p-Smad and anti-total Smad antibodies showed that SY-LB-35 and SY-LB-57 mimicked BMP2 by increasing Smad phosphorylation levels. Quantification of p-Smad levels compared to control confirms significant upregulation of p-Smad following 30-min stimulation with BMP2 (195%, ****p < 0.0005), SY-LB-35 (0.01 µM: 150%, 0.1 µM: 170%, *p < 0.05; 1 µM: 282%, ****p < 0.0005; 10 µM: 188%, **p < 0.01) and SY-LB-57 (0.01 µM: 234%, ***p < 0.001; 0.1 µM: 316%, 1 µM: 276%, 10 µM: 357%, ****p < 0.0005). Levels of p-Smad were normalized to total Smad levels and are expressed as a percent of control (mean ± SEM; n = 3). The original, uncropped blots are presented in Supplemental Fig. S4. (**B**–**E**). Serum-starved C2C12 cells grown on PDL-coated glass coverslips were treated with BMP2 (50 ng/mL), 1 µM SY-LB-35 or 1 µM SY-LB-57 for 30 min. Unstimulated cells served as the negative control. The cultures were stimulated, fixed, and labelled with phospho-specific anti-Smad primary antibodies and Cy3-conjugated secondary antibodies (red). The coverslips were mounted in medium containing DAPI to label nuclei (blue). Control cells (**B**) have low levels of p-Smad labelling in the nucleus. Stimulation with BMP2 (**C**) resulted in a drastic increase in the level of phosphorylated Smad localized to the nucleus. Both SY-LB-35 (**D**) and SY-LB-57 (**E**) stimulated robust increases in p-Smad levels as well as nuclear translocation of phosphorylated Smads. The merged panels show diffuse p-Smad in the cytoplasm in control cells, whereas the stimulated cells illustrate the complete overlap of p-Smad labelling and the DAPI stain (n = 3; scale = 10 µm).

To investigate whether the new indolyl-benzimidazole compounds activate BMP receptor-dependent signaling pathways, the levels of p-Smad in serum-starved C2C12 cells, treated for 30 min with SY-LB-35 and SY-LB-57, were assessed by Western blot analysis. Whole cell lysates of treated C2C12 cells were probed for p-Smad using phospho-specific anti-Smad antibodies. p-Smad levels were normalized against total Smad levels. BMP2-stimulated cells served as positive control and unstimulated cells helped to establish the baseline levels of p-Smad. BMP2 (50 ng/mL) significantly increased p-Smad levels over control, unstimulated cells (95% over control; ****p < 0.0005; Fig. 5A and Supplemental Fig. S4). SY-LB-35 and SY-LB-57 (0.01–10 µM) strongly increased the phosphorylation of Smad proteins at all concentrations tested by 50% to 257% over control (****p < 0.0005, ***p < 0.001; **p < 0.01; *p < 0.05; Fig. 5A).

To establish that SY-LB-35 and SY-LB-57 also stimulate the translocation of phosphorylated Smad to the nucleus, immunofluorescent labeling was performed. Serum-starved C2C12 cells grown on poly-D-lysine (PDL)-coated coverslips were stimulated with BMP2 (50 ng/mL), SY-LB-35 (1 µM) or SY-LB-57 (1 µM) for 30 min. The cultures were fixed and labelled with anti-p-Smad antibodies (red) and DAPI (blue), which stains the nuclei (Fig. 5B–E). In control, unstimulated C2C12 cells, low levels of diffuse, cytoplasmic p-Smad labeling were detected (Fig. 5B). Treatment of C2C12 cells with SY-LB-35 (Fig. 5D) and SY-LB-57 (Fig. 5E) induced the translocation of p-Smad into the nucleus similar to that induced by BMP2 (Fig. 5C). Thus, like BMP2, SY-LB-35 and SY-LB-57 not only strongly stimulated Smad phosphorylation but also prompted p-Smad nuclear translocation.

### SY-LB-35 and SY-LB-57 induce robust increases in PI3K/Akt signaling

**Figure 6:**
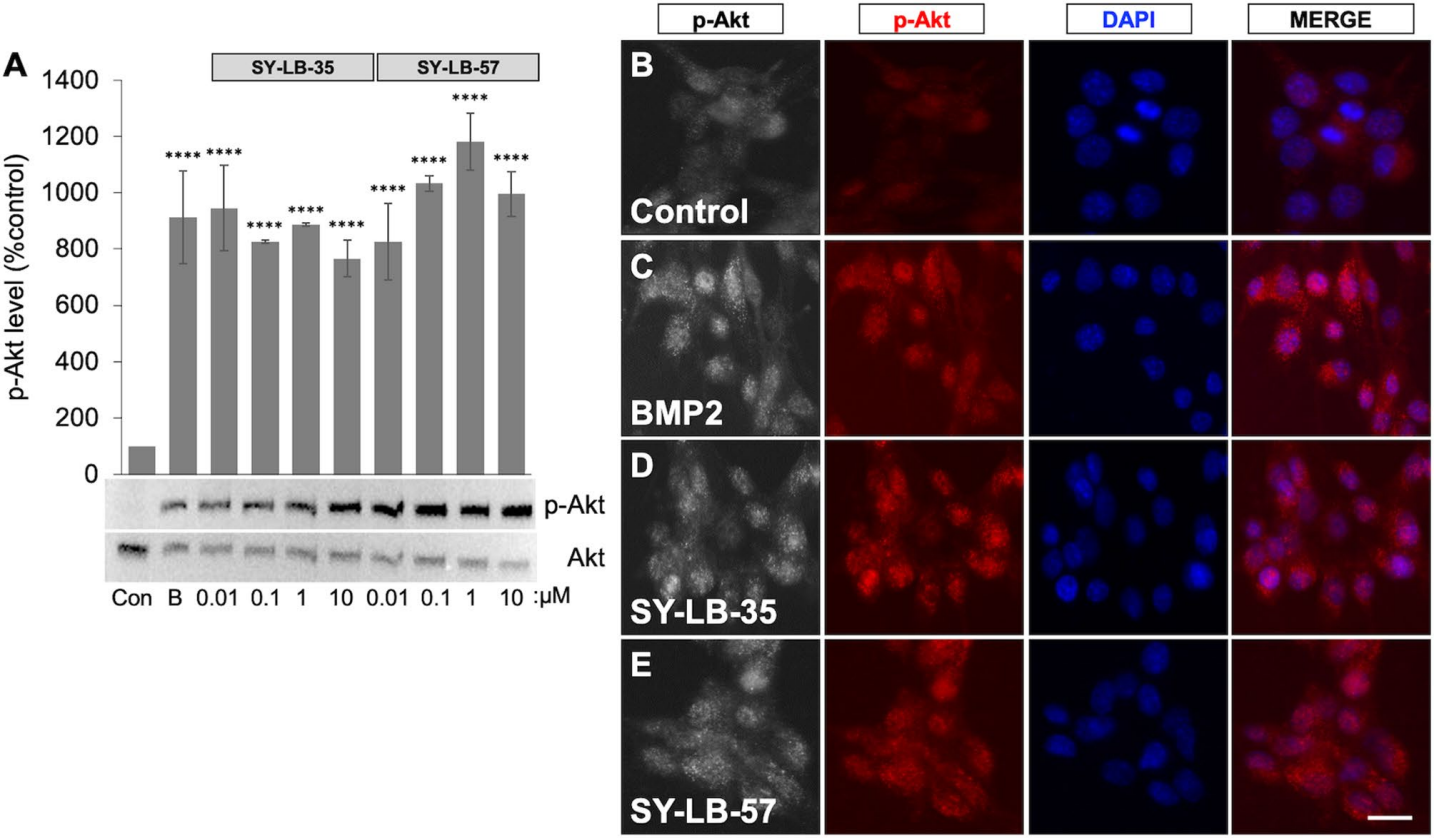
Phosphorylation and cytoplasmic distribution of p-Akt induced by SY-LB-35 and SY-LB-**57. (A**) Western Blot analysis using anti-p-Akt and anti-total Akt antibodies was performed on whole cell lysates of serum-starved C2C12 cells treated with 50 ng/mL BMP2 as a positive control, SY-LB-35 (0.01–10 µM) or SY-LB-57 (0.01–10 µM) for 15 min. Quantification of p-Akt levels with respect to control, untreated cells demonstrates that BMP2 (913%, ****p < 0.0005), SY-LB-35 (0.01 µM: 945%, 0.1 µM: 826%, 1 µM: 887%, 10 µM: 766%, ****p < 0.0005) and SY-LB-57 (0.01 µM: 827%, 0.1 µM: 1033%, 1 µM: 1181%, 10 µM: 995%, ****p < 0.0005) caused significant stimulation of Akt phosphorylation after 15 min in C2C12 cells. Levels of p-Akt were normalized to total Akt levels and are expressed as a percent of control (mean ± SEM; n = 3). The original, uncropped blots are presented in Supplemental Fig. S5. (**B**–**E**). Serum-starved C2C12 cells grown on PDL-coated glass coverslips were treated with BMP2 (50 ng/mL) as positive control, and SY-LB-35 or SY-LB-57 (1 µM) for 15 min. Unstimulated cells were used as the negative control. The cultures were stimulated, fixed, and labelled with phospho-specific anti-Akt primary antibodies and Cy3-conjugated secondary antibodies (red). The coverslips were mounted in medium containing DAPI to label nuclei (blue). Control cells (**B**) have low levels of p-Akt labelling in the cytoplasm. Stimulation with BMP2 (**C**) resulted in a drastic increase in the level of phosphorylated Akt localized to the cytoplasm. SY-LB-35 (**D**) and SY-LB-57 (**E**) stimulated increases in p-Akt levels as well as cytoplasmic translocation of phosphorylated Akt after 15 min of treatment in C2C12 cells. The merged panels illustrate that the increase in p-Akt labelling does not completely overlap with the nuclear DAPI stain (n = 3; scale = 10 µm).

To ascertain whether these novel indolyl-benzimidazole compounds can also activate non-canonical BMP-related pathways, stimulation of the PI3K/Akt pathway was assessed by first monitoring changes in the level of p-Akt in response to SY-LB-35 and SY-LB-57. Serum-starved C2C12 cells were stimulated with BMP2 (50 ng/mL) or increasing concentrations of SY-LB-35 and SY-LB-57 (0.01–10 µM) for 15 min. Western blot analysis of the treated C2C12 whole cell lysates using phospho-specific anti-Akt antibodies showed that BMP2 triggered phosphorylation of Akt significantly (813% over control; ****p < 0.0005; Fig. 6A and Supplemental Fig. S5). Correspondingly, SY-LB-35 and SY-LB-57-treated cell lysates showed a highly significant, robust increase in p-Akt levels at all tested concentrations (667–1081% over control, ****p < 0.0005, Fig. 6A). Immunofluorescent labeling with anti-p-Akt antibodies in unstimulated C2C12 cells showed low level, diffuse p-Akt labeling in the cytoplasm (Fig. 6B). In contrast, 1 µM SY-LB-35 (Fig. 6D) and 1 µM SY-LB-57 (Fig. 6E) induced robust, punctate, cytoplasmic p-Akt labeling that was indistinguishable from the labeling in cells treated with 50 ng/mL BMP2 (Fig. 6C). These results provide strong evidence that SY-LB-35 and SY-LB-57 activate the PI3K/Akt pathway and direct the cytoplasmic localization of p-Akt.

**Figure 7:**
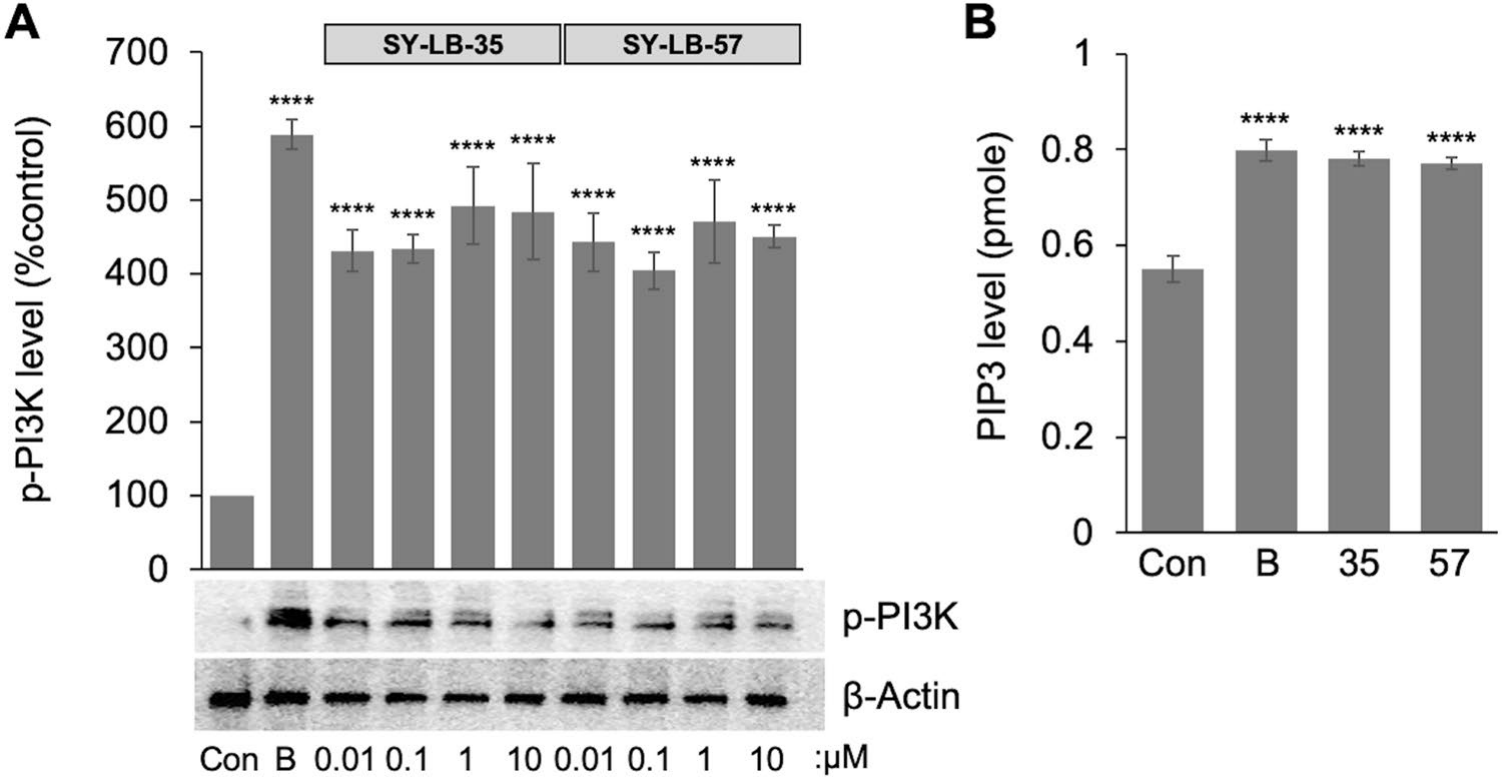
SY-LB-35 and SY-LB-57 stimulate the phosphorylation and activation of PI3K. **(A)** Western Blot analysis using anti-p-PI3K and anti-β-actin antibodies was performed on whole cell lysates of C2C12 cells treated with 50 ng/mL BMP2 as positive control, SY-LB-35 (0.01–10 µM) or SY-LB-57 (0.01–10 µM) for 15 min. Quantification of p-PI3K levels with respect to control, untreated C2C12 cells demonstrates that BMP2 (589%, ****p < 0.0005), SY-LB-35 (0.01 µM: 431%, 0.1 µM: 434%, 1 µM: 492%, 10 µM: 484%, ****p < 0.0005) and SY-LB-57 (0.01 µM: 443%, 0.1 µM: 404%, 1 µM: 471%, 10 µM: 451%, ****p < 0.0005) caused significant increases in p-PI3K levels after 15 min. Levels of p-PI3K were normalized to β-actin levels and are expressed as a percent of control (mean ± SEM; n = 3). The original, uncropped blots are presented in Supplemental Figure S6. (**B**) Serum-starved C2C12 cells were stimulated with SY-LB-35 and SY-LB-57 at 10 µM for 15 min. BMP2 (50 ng/mL) was used as a positive control. Whole cell lysates were prepared and PI3K was immunoprecipitated from the samples and PI3K activity was assessed using a PI3K ELISA. The amount of the product, PIP_3_, produced by PI3K enzyme was significantly higher in BMP2- (0.79 pmol), SY-LB-35- (0.77 pmol) and SY-LB-57-treated samples (0.77 pmol) compared to control (0.61 pmol; ****p ˂ 0.0005, n = 3). The optical density of all samples was measured at 450 nm and the enzyme activity was expressed as the amount of product (PIP_3_ level) generated in each sample by the PI3K per minute of reaction.

To directly assess the activation of the PI3K enzyme, Western blot analysis was carried out on C2C12 cell whole cell lysates after a 15-min stimulation with 0.01–10 µM SY-LB-35 or SY-LB-57. SY-LB-35 and SY-LB-57 stimulated significant increases in the level of PI3K phosphorylation at 15 min at all concentrations tested (> 300% increase, ****p < 0.0005; Fig. 7A and Supplemental Fig. S6). Thus, the novel compounds appear to directly affect the phosphorylation of the PI3K enzyme.

To investigate whether the novel benzimidazoles directly regulate PI3K enzymatic activity, serum-starved C2C12 cells were stimulated with 50 ng/mL BMP2, 10 µM SY-LB-35, or 10 µM SY-LB-57 for 15 min and whole cell lysates were prepared. PI3K enzyme was immunoprecipitated and processed in a PI3K ELISA (Fig. 7B). A standard curve of known concentrations of PIP_3_ versus absorbance was plotted and used to determine the amount of PIP_3_ generated from a PIP_2_ substrate by active PI3K enzyme immunoprecipitated from the treated cell samples (Supplemental Fig. S7). The enzyme activity is expressed as the amount of product (PIP_3_) generated in each sample by the PI3K enzyme per minute of reaction. The results of PI3K ELISA assay revealed that the amount of the PIP_3_ produced by PI3K enzymatic activity was significantly higher in BMP2- (30%), SY-LB-35- (26%) and SY-LB 57-treated samples (26%) compared to control, untreated cells (****p < 0.0005; Fig. 7B) confirming the direct activation of PI3K in response to SY-LB-35 and SY-LB-57.

**Figure 8:**
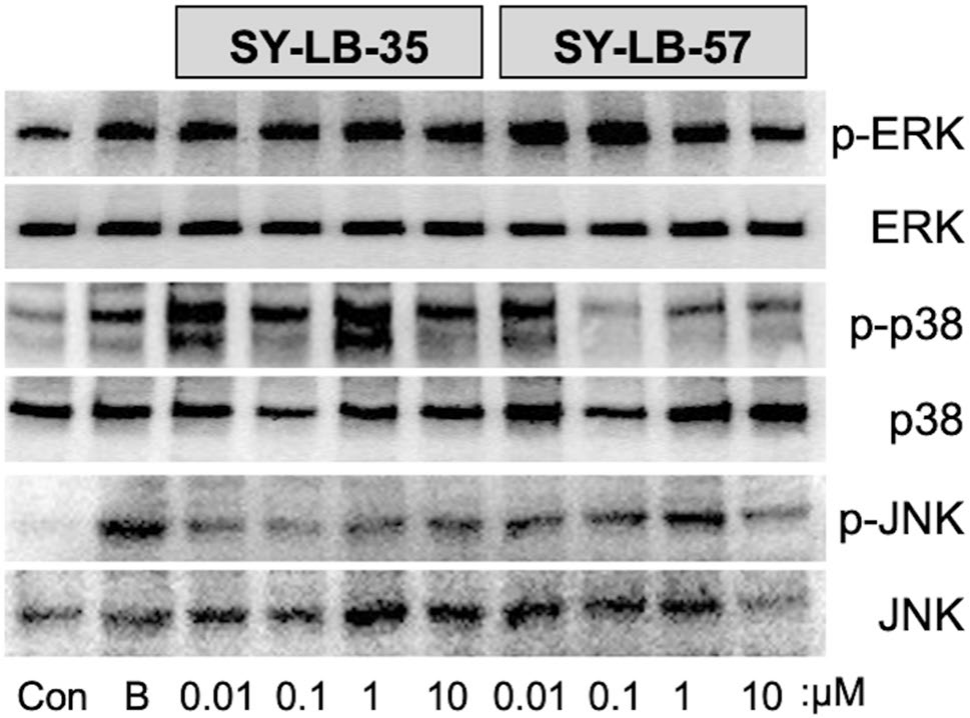
Activation of ERK, p38 and JNK phosphorylation by SY-LB-35 and SY-LB-57. (**A**) Western Blot analysis was performed on whole cell lysates of serum-starved C2C12 cells stimulated by SY-LB-35 or SY-LB-57 (0.01–10 µM) for 15 min. BMP2 (50 ng/mL) served as the positive control. Blots were probed with antibodies against p-ERK, ERK, p-p38, p38, p-JNK and JNK. SY-LB-35 and SY-LB-57 stimulated increases in phosphorylation of all three intracellular signaling targets at all concentrations tested (n = 3). The original, uncropped blots are presented in Supplemental Fig. S8 (ERK blots), S9 (p38 blots) and S10 (JNK blots).

To determine whether SY-LB compounds can activate additional non-canonical BMP-stimulated signaling pathways, C2C12 were stimulated with BMP2 (50 ng/mL) as a positive control and 0.01–10 µM SY-LB-35 or SY-LB-57 for 15 min. Whole cell lysates were prepared from the treated cultures for Western blot analysis. SY-LB-35 and SY-LB-57 stimulated increases in the levels of p-ERK, p-p38 and p-JNK after 15 min of stimulation (Fig. 8 and Supplemental Figs. S8–S10). Taken together, these data demonstrate that SY-LB-35 and SY-LB-57 activate the major downstream targets of non-canonical BMP receptor activation including PI3K/Akt, ERK, p38 and JNK.

### SY-LB-35 and SY-LB-57 induce a shift in cell cycle in favor of proliferation

**Figure 9:**
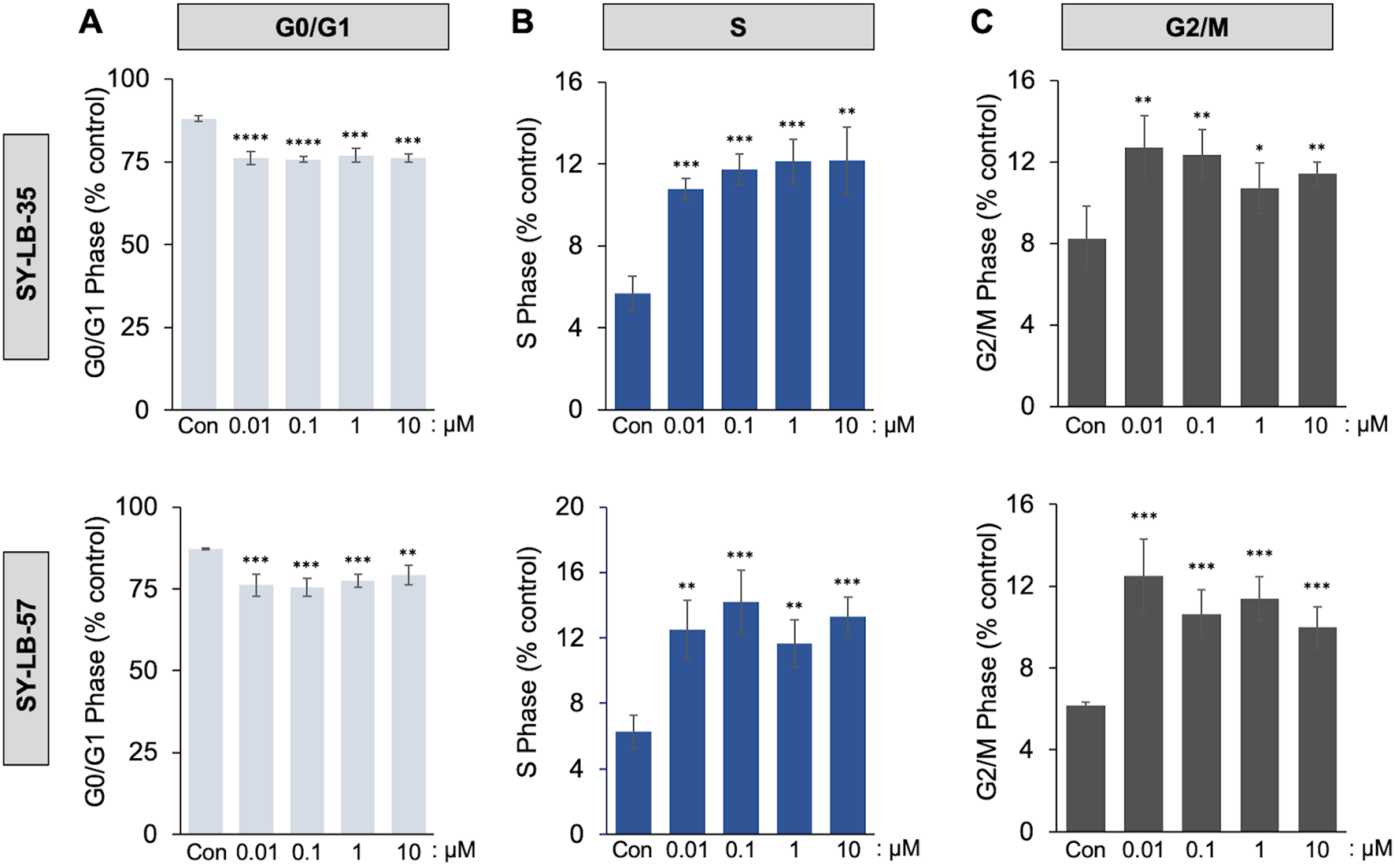
Shifts to S and G2/M phases of the cell cycle induced by SY-LB-35 and SY-LB-57. Serum-starved C2C12 cells were treated with SY-LB-35 (top row) and SY-LB-57 (bottom row) for 24 h. An equal number of cells (~ 10^6^ cells per treatment) was collected for flow cytometry analysis. Quantitative analysis of the distribution or proportion of cells in each phase was carried out with at least 20,000 cells per sample. Cell cycle analysis demonstrated that SY-LB-35 and SY-LB-57 (0.01–10 µM) caused significant shifts in the phases of the cell cycle from G0/G1 phases **(A**) to S (**B**) and G2/M (**C**) phases compared with control, untreated C2C12 cells. SY-LB-35: Con (G0/G1: 88%, S: 6%, G2/M: 8%); 0.01 µM (G0/G1: 76%, S: 11%, G2/M: 13%); 0.1 µM (G0/G1: 76%, S: 12%, G2/M: 12%); 1 µM (G0/G1: 77%, S: 12%, G2/M: 11%); 10 µM (G0/G1: 76%, S: 12%, G2/M: 11%); SY-LB-57: Con (G0/G1: 87%, S: 6%, G2/M: 6%); 0.01 µM (G0/G1: 76%, S: 13%, G2/M: 13%); 0.1 µM (G0/G1: 76%, S: 14%, G2/M: 11%); 1 µM (G0/G1: 77%, S: 12%, G2/M: 11%); 10 µM (G0/G1: 79%, S: 13%, G2/M: 10%). Each bar represents mean ± SEM (n = 3) with each experiment carried out in triplicate (****p < 0.0005; ***p < 0.001; **p < 0.01; *p < 0.05).

In light of the large increases in cell viability and the stimulation of growth-promoting pathways like PI3K/Akt, ERK and p38 by the novel small molecules, the proliferative effects of SY-LB-35 and SY-LB-57 were explored using cell cycle analysis. C2C12 cells were starved in SS medium followed by 24-h treatment with 0.01–10 µM SY-LB-35 or SY-LB-57. The distribution of cell cycle phases was analyzed with respect to the control, untreated cells (Fig. 9). Flow cytometry analysis revealed a significant increase in percentage of cells present in S phase (****p < 0.0005; ***p < 0.001; **p < 0.01; *p < 0.05; Fig. 9B) and G2/M phases (***p < 0.001; **p < 0.01; *p < 0.05; Fig. 9C) in response to SY-LB-35 and SY-LB-57 compared to control, untreated cells. Furthermore, the percentage of cells in G0/G1 phase decreased with respect to control following the treatment of C2C12 cells with the indolyl-benzimidazole compounds (****p < 0.0005; ***p < 0.001; **p < 0.01; Fig. 9A). Thus, SY-LB-35 and SY-LB-57 at all tested concentrations shifted the cell cycle in C2C12 cells toward proliferation causing a significant decline in the population of cells in G0/G1 phase and a sharp increase in the percentage of cells present in proliferative phases of the cell cycle (S and G2/M).

### Smad stimulation by SY-LB-35 and SY-LB-57 depends on type I BMP receptor activity

**Figure 10:**
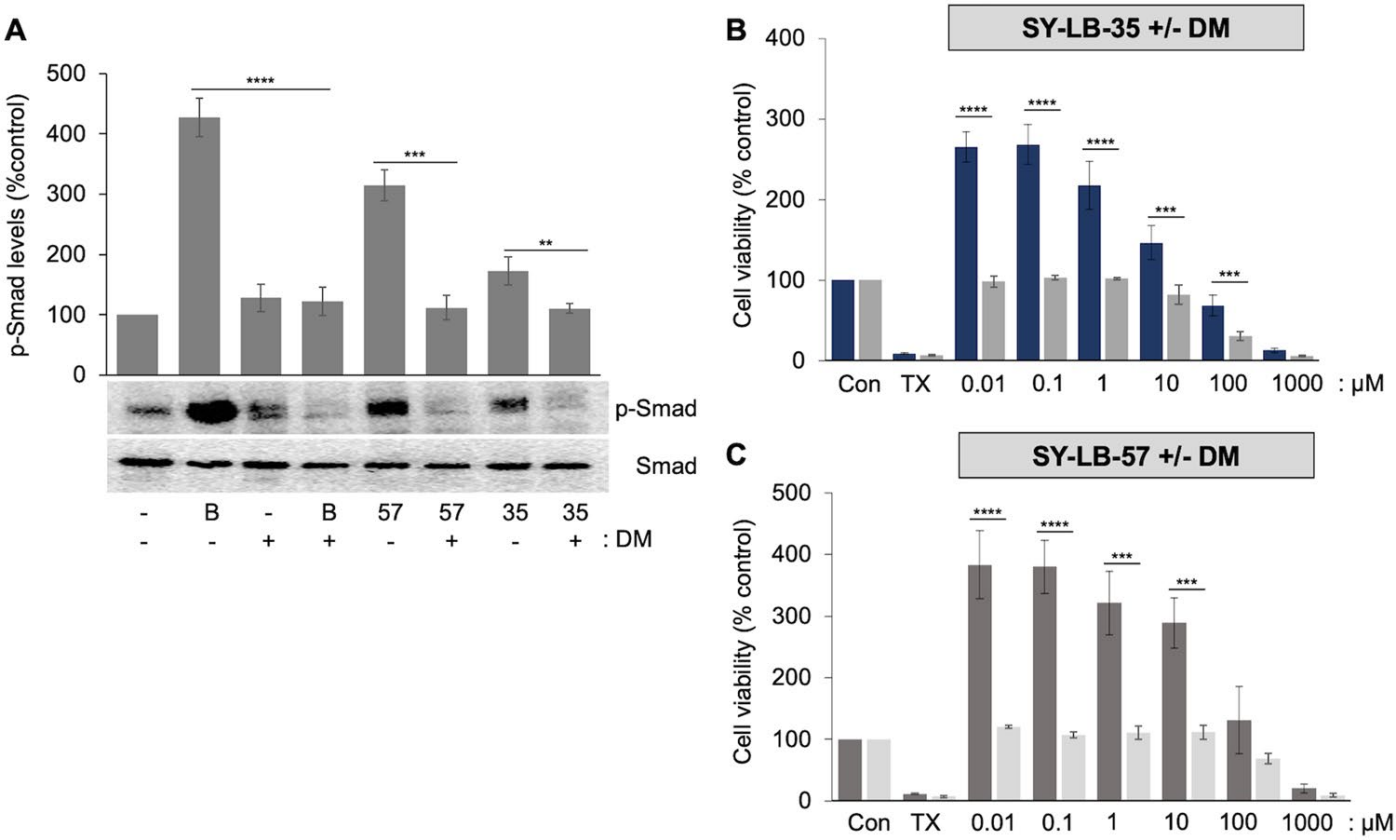
Increases in p-Smad by SY-LB-35 and SY-LB-57 is mediated by type I BMP receptor activity. (**A**) Western blot analysis using anti-p-Smad and anti-total Smad antibodies of serum-starved C2C12 whole cell lysates stimulated with BMP2 (50 ng/mL) as a positive control and 1 µM SY-LB-35 or SY-LB-57 in presence or absence of an inhibitor of type I BMP receptor activity, Dorsomorphin (DM; 10 µM), for 30 min. Pretreatment of C2C12 cells with DM for 1 h inhibited increases in Smad phosphorylation stimulated by BMP2 (DM(−) 428% versus DM(+) 122%, ****p < 0.0005), SY-LB-35 (DM(−) 315% versus DM(+) 112%, ***p < 0.001) and SY-LB-57 (DM(−) 172% versus DM(+) 111%, **p < 0.01). The level of p-Smad was normalized to Smad levels. Data are expressed as mean ± SEM (n = 3). The original, uncropped blots are presented in Supplemental Fig. S11. (**B**,**C**). C2C12 cells were seeded in a 96-well plate, starved for 16 h, and stimulated with SY-LB-35 (**B**) or SY-LB-57 (**C**) (0.01–1000 µM) in presence or absence of DM (10 µM) for 24 h. Triton X-100 (125 µM) was used as negative control. DM blocked all increases in cell viability induced by SY-LB-35 (0.01 µM: DM(−) 266% versus DM(+) 98%; 0.1 µM: DM(−) 269% versus DM(+) 103%; 1 µM: DM(−) 218% versus DM(+) 102%, ****p < 0.0005; 10 µM: DM(−) 147% versus DM(+) 82%; 100 µM: DM(−) 69% versus DM(+) 30%, ***p < 0.001) and SY-LB-57 (0.01 µM: DM(−) 384% versus DM(+) 120%; 0.1 µM: DM(−) 380% versus DM(+) 107%, ****p < 0.0005; 1 µM: DM(−) 321% versus DM(+) 111%; 10 µM: DM(−) 289% versus DM(+) 111%, ***p < 0.001). The data are expressed as mean ± SEM with each experiment conducted in triplicate (n = 3).

To examine the contribution of type I BMP receptor activity to SY-LB-35- and SY-LB-57-stimulated Smad signaling, serum-starved C2C12 cells were stimulated with SY-LB-35 or SY-LB-57 at 1 µM for 30 min in presence or absence of Dorsomorphin (DM; a non-selective inhibitor of type I BMP receptors; 10 µM). Total whole cell lysates were prepared and used for Western blot analysis of Smad phosphorylation using anti-p-Smad antibodies. Pretreatment of C2C12 cells with DM completely prevented the increase in p-Smad levels induced by SY-LB-35 and SY-LB-57 suggesting that these select indolyl-benzimidazole compounds act through a mechanism similar to endogenous BMP ligands, like BMP2, which rely upon type I BMP receptor activity (****p < 0.0005; ***p < 0.001; **p < 0.01; Fig. 10A and Supplemental Fig. S11).

To resolve whether the SY-LB-35 or SY-LB-57 compound requires type I BMP receptor activity for the induction of cell viability in C2C12 cells, cell viability assays were carried out in the presence or absence of 10 µM DM. After 24 h, the presence of DM led to complete inhibition of the cell viability increases stimulated by SY-LB-35 and SY-LB-57 compared to cell viability responses in the absence of DM (****p < 0.0005; ***p < 0.001; **p < 0.01; *p < 0.05; Fig. 10B,C). Thus, the novel benzimidazoles, SY-LB-35 and SY-LB-57, appear to stimulate both canonical BMP signaling and robust increases in cell viability through a process dependent on the activity of type I BMP receptors.

## Discussion

There has been growing interest in identifying small molecules as useful modulators of BMP signaling. Classical approaches involve HTS of small molecule libraries to identify promising lead compounds. Based on the available literature, two privileged heterocyclic scaffolds that are components of reported BMP signaling modulators were identified as candidates for further exploration. PD407824 is a carbazole derivative and contains an indole structure^22^. A series of compounds reported by Bradford et al*.* contain a benzimidazole as the heterocyclic motif and shown to be essential for activity^13^. Compared to recombinant proteins, such synthetic small molecules provide several advantages as therapeutic agents. Small molecules are typically more stable, easily accessible via chemical synthesis, and pharmacological properties can be optimized via medicinal chemistry efforts. The previously reported small molecules identified as BMP-like show relatively low activity and fail to fully activate BMP signaling. Thus, there is still a strong need to identify efficient BMP agonists that could replace approaches currently using less stable, inefficient, proteinaceous preparations of recombinant BMPs to treat BMP-related pathologies. Herein, the synthesis and evaluation of two novel compounds, SY-LB-35 and SY-LB-57 (Fig. 2), is reported. The design for these compounds came from the observation that indole and benzimidazole motifs are found in reported BMP modulators^13,22–24^ and are known to be privileged scaffolds in the drug discovery field^26,28,29^. A recently developed synthetic methodology within the Yoganathan lab provided an ideal opportunity to design and synthesize a hybrid molecule that contains the indole and benzimidazole scaffolds. These compounds were synthesized from commercially available carboxylic acid precursors and the synthesis was carried out in as high as 1 g scale. As the precursors are readily available and the synthesis can be executed in multi-gram scale, it is possible to do extensive biological evaluation both in vitro and in vivo. Since PD407824 (compound **1;** Fig. 1) has a hydroxyl group at the 5-postion of the indole structure, we designed the two compounds to contain the same oxygenation pattern on the indole motif. SY-LB-35 has a hydroxyl group at the 5-position of the indole unit (compound **10**; Fig. 2) and SY-LB-57 has a methoxy group at the same position on the indole motif (compound **11**; Fig. 2). These two functionalities provide different physiochemical properties to these analogs and likely different types of intermolecular interactions to a potential protein target. It is expected that SY-LB-35 will be slightly more hydrophilic, in comparison to SY-LB-57. Additionally, having the hydroxyl handle on the indole unit (SY-LB-35) provides future opportunities to explore a larger library of compounds through medicinal chemistry efforts. Based on our own research, we have established a synthetic method that allows selective derivatization of the benzimidazole or the indole unit via *N-*alkylation chemistry for further investigation of these hybrid compounds^25^. Moreover, the initial biological evaluation revealed promising cell viability data, demonstrating that these new compounds are likely stimulating cell growth in C2C12 mouse myoblasts. A concentration–response analysis showed that these two compounds are essentially non-toxic to C2C12 cells at concentrations well above 100 µM. Indeed, the calculated IC_50_ values for SY-LB-35 and SY-LB-57 are 401 µM and 808 µM, respectively.

Previous efforts to stimulate BMP signaling pathways has led to the discovery of a few small molecules, which failed to fully activate the canonical Smad pathway. The BMP sensitizer, PD407824, did not induce Smad phosphorylation but, rather interestingly, reduced p-Smad levels in the absence of BMP. However, in the presence of BMP, PD407824 slightly increased p-Smad levels over BMP alone through an indirect mechanism involving inhibition of Smad2/3, which serve as R-Smads for TGFβ ligands^22^. HTS for agonists of BMP signaling introduced a series of compounds, typified by sb4, which increase p-Smad levels twofold over control compared to 11-fold for BMP4 in 30-min stimulation assays. Furthermore, sb4 appears to induce Smad phosphorylation through a mechanism that is independent of type I BMP receptors^13^.

In the current study, SY-LB-35 and SY-LB-57 at sub-micromolar concentrations stimulated robust increases in p-Smad levels in C2C12 cells after 30 min (100–250% increase over control; Fig. 5A). In comparison, BMP2 at a relatively high concentration of 50 ng/mL caused ~ 100% increase in level of phosphorylated Smads in the same cells demonstrating the efficacy of these novel indolyl-benzimidazoles. Moreover, inhibition of type I BMP receptor activity by DM indicated that Smad phosphorylation by these compounds relies on the activity of type I BMP receptors. Furthermore, immunofluorescence analysis of C2C12 cells treated with these compounds confirmed strong translocation of p-Smad into the nucleus, which was indistinguishable from the response to BMP2 (Fig. 5C–E).

SY-LB-35 and SY-LB-57 also significantly boosted the levels of p-Akt and induced cytoplasmic distribution of p-Akt in C2C12 cells (Fig. 6). Previous examination of type II BMP receptor-dependent regulation of PI3K/Akt activity revealed that BMP-evoked Akt phosphorylation was inhibited when ActRIIA expression was deficient^30^. Moreover, specific type II BMP receptor subunits, ActRIIA and BMPR2, were required for BMP-induced growth cone collapse in developing spinal neurons and for chemotaxis of monocytes^21,30^. In the present study, assessment of BMP signaling by SY-LB-35 and SY-LB-57 suggests that these compounds might induce Akt activation through type II BMP receptors as well. Taken together, these data strongly support the idea that SY-LB-35 and SY-LB-57, likely working through type I BMP receptors, can mirror the activity of recombinant BMPs to trigger BMP signaling pathways and, thus, appear to act as BMP agonists.

Type I BMP receptors initiate intracellular signaling by phosphorylating specific R-Smads, which ultimately leads to transcriptional regulation of target genes^18^. Stimulation of the PI3K/Akt pathway by BMPs has been biochemically and functionally linked to type II BMP receptor-dependent signaling^19,21,30^. How dimeric BMPs stimulate these distinct pathways through interaction with the tetrameric BMP receptor complex is an unanswered question in the BMP receptor field.

Early evidence of small molecules activating BMP receptors was obtained from the study of immunosuppressants such as sirolimus (rapamycin) and tacrolimus (FK506), which appeared to liberate type I BMP receptors from inhibition by the immunophilin, FKBP12, and promote activation of BMP receptors intracellularly leading to activation of the Smad pathway^5,31^. Further efforts to identify small molecule agonists of BMPs resulted in development of sb-series compounds, which marginally enhanced BMP signaling^13^. sb4-mediated activation of the BMP pathway was resistant to inhibition by the endogenous BMP antagonist, noggin, or by type I BMP receptor inhibitors suggesting that sb4 works through a mechanism that does not involve type I BMP receptor activity^13^. Dependence on type I BMP receptor activity observed in this study suggests a more direct activation of BMP signaling by SY-LB-35 and SY-LB-57 resulting in increases in p-Smad levels on par with BMP-evoked activity. Inhibition of Smad signaling in the presence of DM showed that these indolyl-benzimidazole compounds may interact directly with type I BMP receptors or with a receptor-associated component like FKBP12. Moreover, pretreatment of C2C12 cells with DM not only decreased p-Smad levels but also completely blocked SY-LB-35- and SY-LB-57-induced increases in cell viability suggesting that these indolyl-benzimidazole compounds might functionally regulate BMP receptor activity. Furthermore, the ability of SY-LB-35 and SY-LB-57 to also stimulate robust phosphorylation of PI3K and Akt suggests that these compounds may regulate the activity of the whole tetrameric receptor complex. It will be important to determine whether and how these novel compounds interact with BMP receptor subunits. Moreover, given the differences in activity observed between SY-LB-35 and SY-LB-57 in Smad, Akt, p38 and JNK phosphorylation and in cell viability assays, it will be interesting to explore how derivatization of the indole or benzimidazole unit might influence the activity of type I or type II BMP receptor-dependent signaling pathways.

Treatment of C2C12 cells with either SY-LB-35 or SY-LB-57 for 15 min significantly upregulated the level of PI3K, Akt, ERK, p38 and JNK phosphorylation. The downstream effector molecules of the PI3K/Akt, ERK and p38 pathways are known to be involved in cellular proliferation^32–34^. Given the increases in cell viability, cell number as well as cell cycle shifts towards S and G2/M phases observed following 24-h treatment with SY-LB-35 and SY-LB-57, it is reasonable to infer that these compounds may mediate proliferation via stimulation of PI3K/Akt, ERK or p38. BMPs are most well-known for their morphogenetic activities, which involves processes of growth, differentiation, and remodeling of tissues^1,2,4,16,18^. Indeed, SY-LB-35 and SY-LB-57 stimulated significant growth of C2C12 cells indicating the morphogenetic potential of these novel indolyl-benzimidazoles.

BMPs regulate key processes during embryonic development, like formation of neural crest cells and patterning of the spinal cord and brain^2,4^. Emerging roles for BMPs have been recognized in maintaining adult tissue homeostasis and deficits in BMP-dependent signaling have been demonstrated to contribute to certain pathologies in adults^35^. Thus far, a few compounds have been introduced as positive regulators of BMP signaling and primarily act as enhancers or sensitizers increasing the responsiveness of the cells to BMPs or stabilizing Smad signling complexes^11,36,37^. In the current study, two novel chemical entities, SY-LB-35 and SY-LB-57, were introduced sharing an indolyl-benzimidazole core, which stimulated canonical and non-canonical BMP signaling pathways and faithfully mimicked recombinant BMP2 signaling activity in C2C12 cells. These compounds demonstrated substantial activation of the canonical Smad-dependent pathway and the Smad-independent, non-canonical PI3K/Akt pathway. Moreover, Smad activation by these compounds was blocked by inhibition of type I BMP receptor activity. Importantly, these compounds not only activated Smad signaling but appear to functionally mimic endogenous BMPs in cell viability and cell cycle assays.

Taken together, the data suggests that the SY-LB-35 and SY-LB-57 are efficient, non-selective activators of BMP receptor-dependent signaling pathways making them strong candidates for developing therapeutics that can robustly activate BMP receptor signaling in human disease conditions in which BMP signaling is defective or lost.

## Materials and methods

### Chemical synthesis

All chemicals and solvents were used without further purification. The two indolyl-benzimidazoles were synthesized using a one-pot synthetic methodology (Fig. 2) based on previous studies^27^. Commercially available indole-2-carboxylic acid derivatives (compounds **6**, **7**) (1 equiv.) and N-diisopropylethylamine (DIPEA) (2.0 equiv.) were dissolved in 10 mL DMF and stirred for 10 min. Then, *O*-benzotriazol-1-yl N,N,N',N'-tetramethyluronium hexafluorophosphate (HBTU) (2.0 equiv.) was added, and the reaction mixture was stirred for another 10 min. Next, *O*-phenylenediamine (1.0 equiv.) was added and stirred for another 4 h to generate compounds **8** and **9**. Thereafter, the reaction was heated under reflux for 6 h to produce the two indolyl-benzimidazoles (compounds **10**, **11**). Chemical reactions were analysed by thin layer chromatography (TLC) with silica gel G as the adsorbent (250 microns) on aluminium backed plates (Agela Technologies, Torrance, CA, USA) and Ultraviolet (UV) light at 254 nm or 365 nm for visualization purposes. The reaction vessel was cooled to room temperature (RT) and the reaction was diluted with water (100 mL) followed by extraction of the product using ethyl acetate (EtOAc). The organic phase was dried over anhydrous sodium sulfate, filtered, and concentrated *in vacuo*. The crude product was further purified by column chromatography using hexanes/EtOAc in increasing polarity up to a 1:1 mixture. The fractions containing the desired product were concentrated and recrystallized in hexanes/EtOAc (1:1) to yield the final product. The compounds were characterized by 1D (^1^H and ^13^C) NMR using a Bruker 400 UltrashieldTM spectrometer (400 MHz) equipped with a z-axis gradient probe (Figures S1 and S2) and LC/MS analysis was performed on single quadrupole Agilent Technologies 1260 infinity series LC/MS (Santa Clara, CA, USA). Characterization data for SY-LB-35 and SY-LB-57 can be found in the Supplemental Information.

### Cell culture and determination of cell concentration

C2C12 mouse myoblast cells (ATCC^®^, Manassas, VA, USA) were maintained in complete growth media (DMEM/10% FBS/1X Penicillin/Streptomycin/Glutamine solution (PSG)) at 37 °C in 5% CO_2_. DMEM media (Dulbecco’s Modified Eagle’s Medium with high glucose and L-glutamine and without sodium pyruvate) was from Caisson Labs (Smithfield, UT, USA). FBS was from Atlanta Biologicals (Flowery Branch, GA, USA). To determine the cell concentration, 18 µL cell suspension was mixed with 2 µL Acridine orange/Propidium iodide stain (Logos Biosystems, Annandale, VA, USA). Next, the cell concentration and percent viability were determined from 10 µL stained cells using the Luna-FL™ Dual Fluorescence Cell Counter from Logos Biosystems. For cell counts following treatment with SY-LB-35 or SY-LB-57, C2C12 cells (500 µL) were seeded into 24-well plates at 1 × 10^5^ cells/mL, grown to 80% confluency, serum-starved for 16–18 h using Serum-Starvation Medium (SS medium: DMEM/1X Penicillin/Streptomycin solution (PS)) and treated for 24 h with the novel compounds. The treated cells were then collected by trypsinization, pelleted at 2000 rpm for 3 min at RT, resuspended and counted.

### Measurement of cell viability

C2C12 cells (100 µL) were seeded in a 96-well plate at 5 × 10^5^ cells/mL and incubated overnight in complete growth media to achieve 80% confluency followed by serum-starvation for 16–18 h. Next, the cells were treated with SS medium alone as a positive control, Triton X-100 (125 µM) as negative control, 1 nM to 1 mM SY-LB-35 or SY-LB-57 or 0.001 ng/mL to 100 ng/mL BMP2 (R&D Systems, Minneapolis, MN, USA) for 24 h. For BMP receptor inhibition, cell viability assays were carried out in presence or absence of Dorsomorphin (DM; 10 µM; Sigma Aldrich, St. Louis, MO, USA), a non-selective inhibitor of type I BMP receptor activity.

Next, reagents from the RealTime-Glo™ MT Cell Viability Assay Kit (Promega, Madison, WI, USA) were diluted in SS medium and used according to manufacturer’s protocol. Cellular luminescence was measured using the FilterMax F5 Multi-mode Microplate Reader (Molecular Devices, San Jose, CA, USA). Data from three independent experiments with individual experiments carried out in triplicate was determined and reported as a percentage of control.

### Treatment of cells and preparation of whole cell lysates

C2C12 cells (3 mL) were seeded into 35 mm dishes at 7.5 × 10^4^ cells/mL in complete growth medium, incubated to reach 80% confluence and serum-starved for 16–18 h. Next, cells were treated with 50 ng/mL BMP2 and increasing concentrations of SY-LB-35 or SY-LB-57 for 15 min, 30 min or 24 h. Thereafter, the cultures were washed with ice-cold 1X TBS for 2 min. Lysis Buffer (Cell Signaling Technology, Danvers, MA, USA) supplemented with 1 mM PMSF (phenylmethylsulphonyl fluoride) was added to the cultures and whole cell lysates were prepared.

### Determination of total protein concentration

The Amido-Schwarz TCA precipitation method was used to estimate the amount of total protein in whole cell lysates^38^. Briefly, whole cell lysates were diluted in dH_2_O, and total soluble protein was precipitated using 60% TCA (Trichloroacetic acid) and 1 M Tris/1% SDS. Precipitated protein was spotted onto 0.45 µm nitrocellulose membranes (Millipore, MA, USA), rinsed with 6% TCA and stained with 0.1% Amido Black in 45% methanol/10% acetic acid/45% dH_2_O. The membranes are rinsed at least 3 times in 90% methanol/2% acetic acid/8% dH_2_O until the membrane background was nearly white. The remaining stained protein was eluted from the membranes in 25 mM NaOH/0.05 M EDTA/50% ethanol. Absorbance of the eluted samples was measured at 630 nm using the Eppendorf BioSpectrometer Basic (Lake Forest, CA, USA). The total protein concentration in the whole cell lysates was determined using a BSA standard curve.

### Western blot analysis

Laemmli sample buffer (4X) was added to the whole cell lysates (20 µg) to a final concentration of 1X and heated at 100 °C for 5 min. The samples were immediately cooled on ice for 5 min and centrifuged at 9500 rpm for 2 min at RT. The samples and the Precision Plus Protein Standard (5 µL; Bio-Rad, Hercules, CA, USA) were loaded on 12% SDS polyacrylamide gels. Protein was separated using the Bio-Rad Mini Protean II Gel Electrophoresis System at 135 V. Meanwhile, 0.2 µm nitrocellulose membranes were pre-incubated in cold 1X Transfer Buffer (10 mM Tris/2.5 mM glycine/20% methanol) for at least 30 min. Next, the separated proteins were transferred at 100 V onto the pre-equilibrated nitrocellulose membranes for 1 h in Transfer Buffer, followed by blocking in 5% BSA/0.1% Tween 20/1X TBS for 30 min. The membranes were incubated overnight at 4 °C with the desired primary rabbit monoclonal antibody (p-Smad1/5(S463/465)/9(S465/467), Smad1 (D5907) XP^®^, p-Akt (Ser473) (D9E) XP^®^, or Akt (pan) (C67E7), p-SAPK/JNK (Thr183/Tyr185), SAPK/JNK, β-actin (Cell Signaling Technologies, Danvers, MA, USA)) or rabbit polyclonal antibody (phospho-PI3 Kinase p85 (Tyr458)/p55 (Tyr199) antibody (Cell Signaling Technologies, Danvers, MA, USA), p-p38 MAPK, p38 MAPK, p-ERK1, ERK1/2) (Abclonal, Woburn, MA, USA)) in Antibody Dilution Buffer (1% BSA/0.1% Tween 20/1X TBS) and then washed with 1X TBST (0.1% Tween 20/1X TBS) three times for 15 min. HRP-conjugated Goat Anti-Rabbit IgG secondary antibody (Santa Cruz Biotechnology, Inc., Dallas, TX, USA) in Antibody Dilution Buffer was added to the membranes for 1 h at RT followed by three 15-min washes with 1X TBST. Finally, the blots were developed using SuperSignal ™ West Femto HRP Substrate solutions (Thermo Scientific, Rockford, IL, USA) and analysed using the Omega Lum™ G Imaging System (San Francisco, CA, USA). Quantification of chemiluminescent signals was carried out using ImageJ (Image Processing and Analysis in Java 1.8.0_112).

### PI3K ELISA assay

C2C12 cells were seeded in 35-mm dishes at 7 × 10^4^ cells/mL and incubated at 37 °C in 5% CO_2_ to reach 80% confluency. The cells were starved for 16–18 h and then stimulated with BMP2 (50 ng/mL) as positive control and SY-LB-35 and SY-LB-57 at 10 µM for 15 min at 37 °C. Cells (~ 10^6^) per treatment were collected to prepare whole cell lysates followed by exposure to three freeze/thaw cycles in liquid nitrogen. The samples were centrifuged and PI3K enzyme was immunoprecipitated with a mouse monoclonal anti-PI3K p55 γ antibody ((E-9), Santa Cruz Biotechnology, Dallas, TX, USA) for 1 h at 4 °C with gentle rocking and the immune complexes were collected overnight on a rocking platform at 4 °C with Dynabeads™ Protein A (Life Technologies, Carlsbad, CA, USA). The next day, the immunoprecipitated PI3K enzyme was collected by centrifugation processed for a PI3K ELISA (Echelon Biosciences, Salt Lake, City, UT, USA) according to the manufacturer’s instructions. Standard solutions of PIP_3_ (0, 0.22, 0.67, 2, 6, 18, and 54 pmol) were added to kinase reactions using recombinant PI3K enzyme (Echelon Biosciences, Salt Lake, City, UT, USA) and used to generate a standard curve for production of PIP_3_. The optical density of the PIP_3_ standards and the reactions containing immunoprecipitated PI3K from treated samples was measured at 450 nm.

### Cell cycle analysis

C2C12 cells (10 mL) were seeded into 100 mm dishes at 1 × 10^6^ cells/mL, grown to 80% confluency, serum starved for 16 h and treated with BMP2 (50 ng/mL), SY-LB-35 or SY-LB-57 (0.01–10 µM) for 24 h. Equal numbers (~ 10^6^) of cells, from each treatment counted as above were collected, fixed with 4% paraformaldehyde (PFA) for 10 min at RT and pelleted at 300 g for 5 min. Cell pellets were washed with Stain buffer (BD Pharmingen, San Diego, CA, USA) followed by permeabilization with 90% ice-cold methanol for 15 min at 4 °C. The cells were again washed with Stain buffer and the pelleted cells were stained with BD Pharmingen Propidium Iodide/RNase Staining Buffer (0.5 mL/10^6^ cells) for 30 min at RT while protected from light. Following two more washes, the pellet was resuspended in 225 µL Stain buffer and the cells were divided into 3 microcentrifuge tubes (75 µL each) for analysis with the Amnis^®^ Flowsight Imaging Flow Cytometer (Austin, TX, USA). IDEAS^®^ software (version 6.2, Seattle, WA, USA) was used to analyse the data files.

### Immunofluorescent labeling

In a 24-well plate, 12 mm glass coverslips were coated for 1 h at RT with 500 µL PDL (0.1 mg/mL/ 0.1 M Boric acid), washed 4 times with cell culture-grade distilled water and sterilized under UV light for 30 min. C2C12 cells (500 µL) were seeded onto the PDL-coated coverslips at a density of 1 × 10^5^ cells/mL, grown to 80% confluency and starved in SS medium for 2 h. Next, the cells were treated with 50 ng/mL BMP2 or 1 µM SY-LB-35 or SY-LB-57 for 15 min (for p-Akt labelling) or 30 min (for p-Smad labelling). Cells were then fixed using ice-cold 4% PFA/PBS for 10 min at RT. Next, the coverslips were washed with ice-cold 1X PBS, transferred to a humidified chamber and incubated (100 µL/coverslip) in Permeabilization solution (0.1% Triton X-100/PBS) for 15 min, then Blocking Buffer (10% heat inactivated goat serum/ 0.3% Triton X-100/PBS) for 30 min. Anti-p-Smad antibodies in Antibody Dilution Buffer (1% BSA/0.1% Tween 20/PBS) were added for 1 h and the coverslips were washed 4 times with 1X PBS. The Cy3-conjugated goat anti-rabbit IgG secondary antibodies (Jackson Immuno Research Labs, West Grove, PA, USA) in Antibody Dilution Buffer were added for 1 h followed by 4 washes with 1X PBS and once with dH_2_O. Finally, the coverslips were mounted on glass microscope slides in Vectashield Mounting Medium containing DAPI (Vector Laboratories, Inc., Burlingame, CA, USA). Images were obtained using a Zeiss Axioplan 200 M upright fluorescent microscope, AxioCam HRm digital camera and AxioVision 4.8.2.0 software.

### Statistical analysis

Significance is defined as *p* ≤ 0.05. Levels of significance are indicated as follows: *p < 0.05, **p < 0.01, ***p < 0.001 and ****p < 0.0005. All statistical analyses were performed with Microsoft Excel using unpaired, two-tailed Student’s t-tests. The IC_50_ values were determined using non-linear regression analysis (Excel) and plotted with GraphPad Prism v.5.0 TM.

## Supplementary Information

Supplementary Information.

## Data Availability

All data generated or analyzed during this study are included in this article and its supplementary information files.
