# Supplementary material for: Discovery of a novel class of benzimidazoles as highly effective agonists of bone morphogenetic protein (BMP) receptor signaling
Source: Sci Rep. 2022 Jul 15;12:12146. doi: 10.1038/s41598-022-16394-x (PMC9287337; doi:10.1038/s41598-022-16394-x)
Supplement: Supplementary file 1 — Supplementary Information. [file 41598_2022_16394_MOESM1_ESM.pdf]

Supplemental Material – Najafi *et al.*

Characterization data for the novel indolyl-benzimidazoles, SY-LB-35 and SY-LB-57.

(5-hydroxy-1H-indol-2-yl)-1H-benzo[d]imidazole (SY-LB-35): Grey solid, 0.19 g, 78 %; R_f 0.203 (1:1/Hexanes:EtOAc); **^{1}H NMR:** (DMSO-d_6, 400 MHz): δ = 12.98 (s, 1H, NH), 12.03 (s, 1H, NH), 7.66 (m, 2H, Ar-H), 7.56 (d, *J*=7.4 Hz, 1H, Ar-H), 7.48 (m, 1H, Ar-H), 7.24 (m, 4H, Ar-H), 7.05 (m, 1H, Ar-H), 3.41 (s, 1H, OH); **^{13}C NMR:** (DMSO-d_6, 100 MHz) δ 146.6, 144.2, 137.7, 135.2, 129.1, 128.3, 123.3, 123.1, 122.2, 121.3, 120.2, 118.9, 112.4, 111.6, 102.1; **LC-MS:** (ESI) *m/z* calculated for $C_{15}H_{12}N_3O[M+H]^+$ 250.0975, observed 250.0979.

2-(5-methoxy-1*H*-indol-2-yl)-1*H*-benzo[*d*]imidazole (SY-LB-57): White solid, 0.46 g, 92%; R_f 0.47 (9:1/CH_2Cl_2:MeOH); **^{1}H NMR:** (DMSO-d_6, 400 MHz): δ = 12.93 (s, 1H, NH), 11.86 (s, 1H, NH), 7.67 (d, *J*=7.2 Hz, 1H, Ar-H), 7.56 (d, 1H, Ar-H), 7.34 (dd, *J*=8.8, 8.8 Hz, 1H, Ar-H), 7.23 (m, 2H, Ar-H), 6.83 (dd, *J*=8.8, 8.8 Hz, 1H, Ar-H), 3.79 (s, 3H, OCH_3); **^{13}C NMR:** (DMSO-d_6, 100 MHz) δ 154.3, 146.7, 144.2, 135.2, 132.9, 129.4, 128.7, 122.9, 122.1, 118.9, 114.9, 114.1, 113.3, 113.2, 111.6, 102.2, 101.9, 55.7; **LC-MS**: (ESI) *m/z* calculated for $C_{16}H_{14}N_3O$ $[M+H]^+$ 264.1131, observed 264.1135.

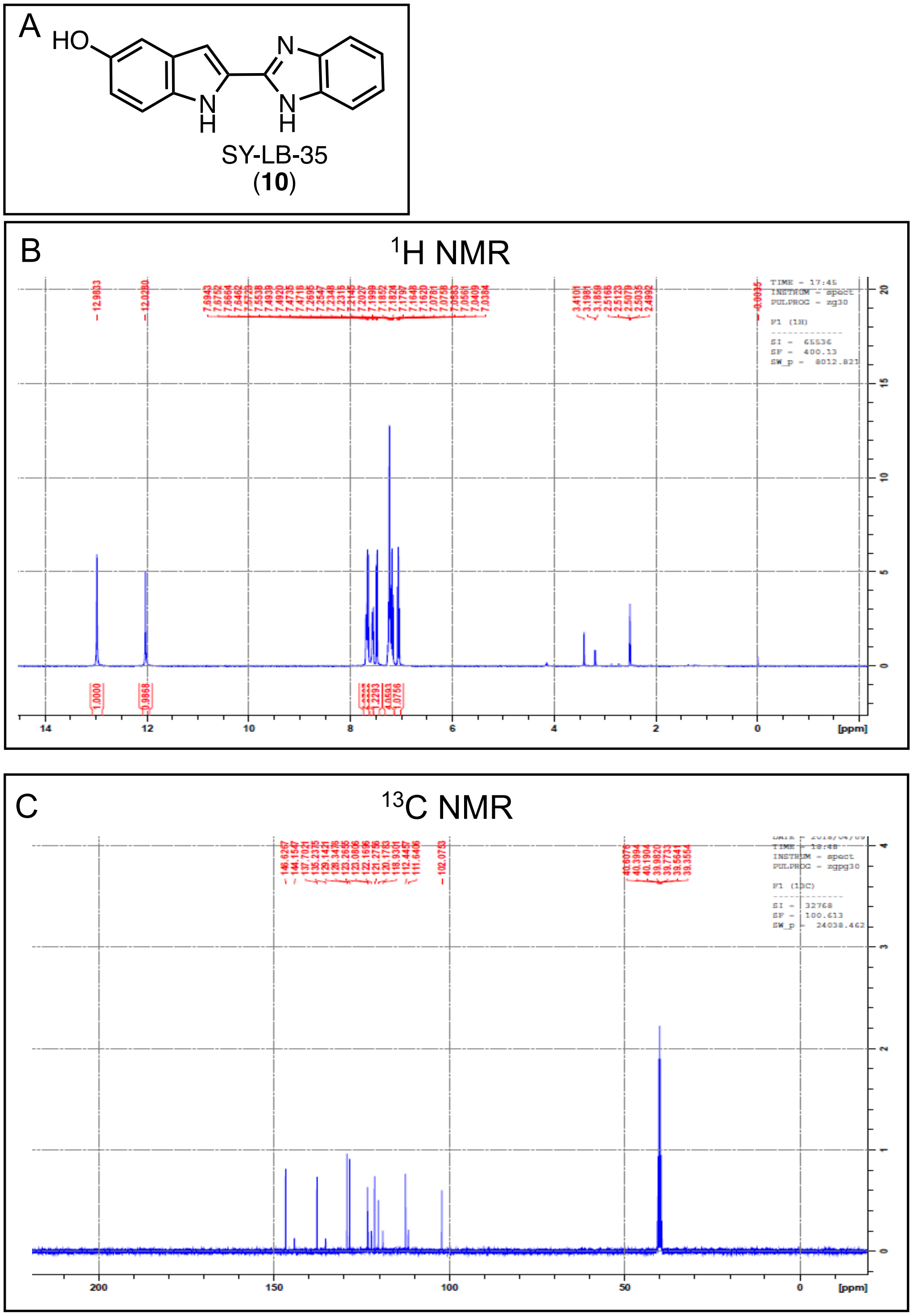

Supplemental Figure S1. ^{1}H and ^{13}C NMR Spectra for SY-LB-35. **(A)** Full structure of SY-LB-35. **(B-C)** The proton **(B)** and carbon **(C)** NMR spectra for SY-LB-35. The samples were dissolved in dimethyl sulfoxide-d_6 for the NMR analysis.

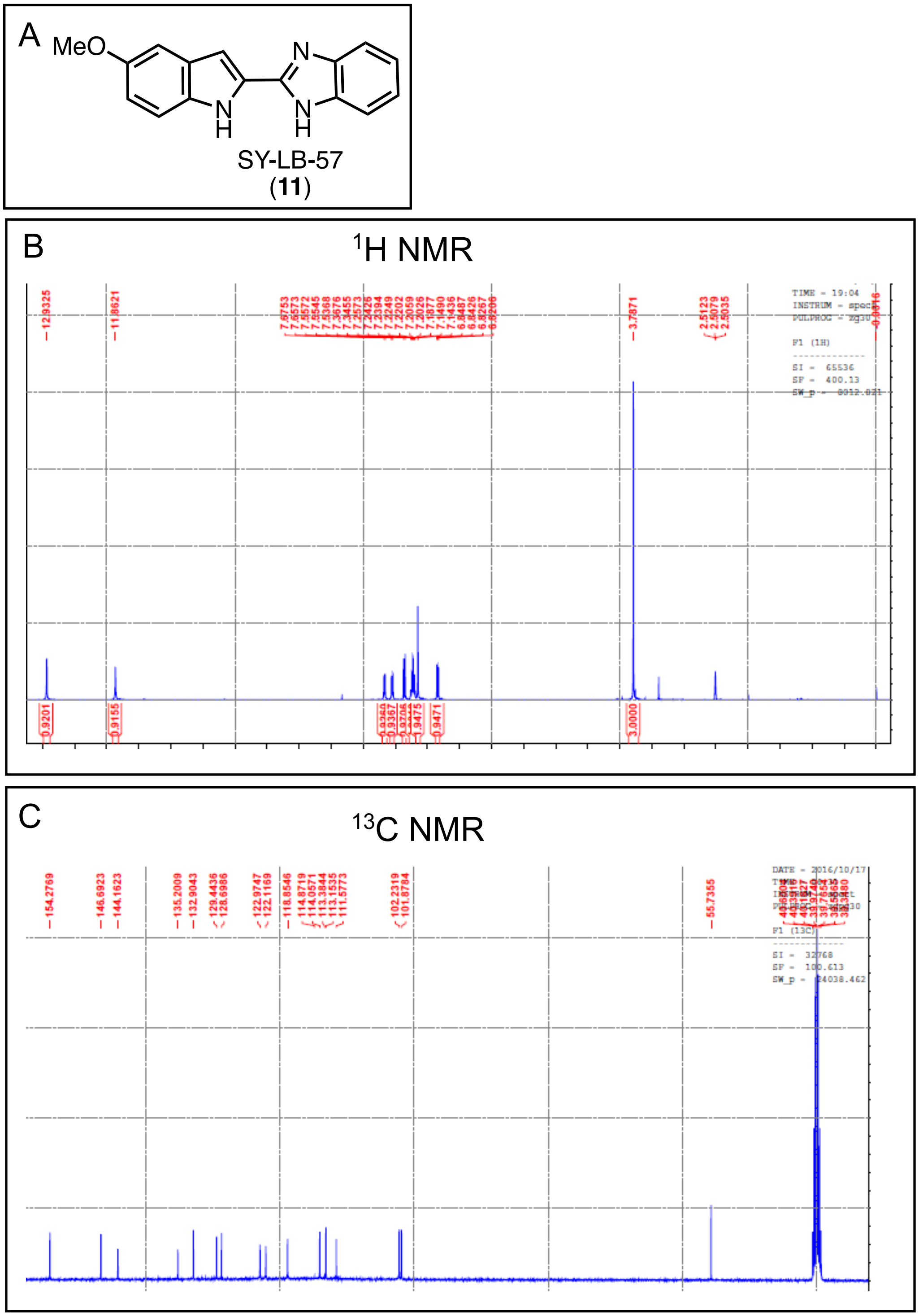

Supplemental Figure S2. ^{1}H and ^{13}C NMR Spectra for SY-LB-57. **(A)** Full structure of SY-LB-57. **(B-C)** The proton **(B)** and carbon **(C)** NMR spectra for SY-LB-57. The samples were dissolved in dimethyl sulfoxide-d_6 for the NMR analysis.

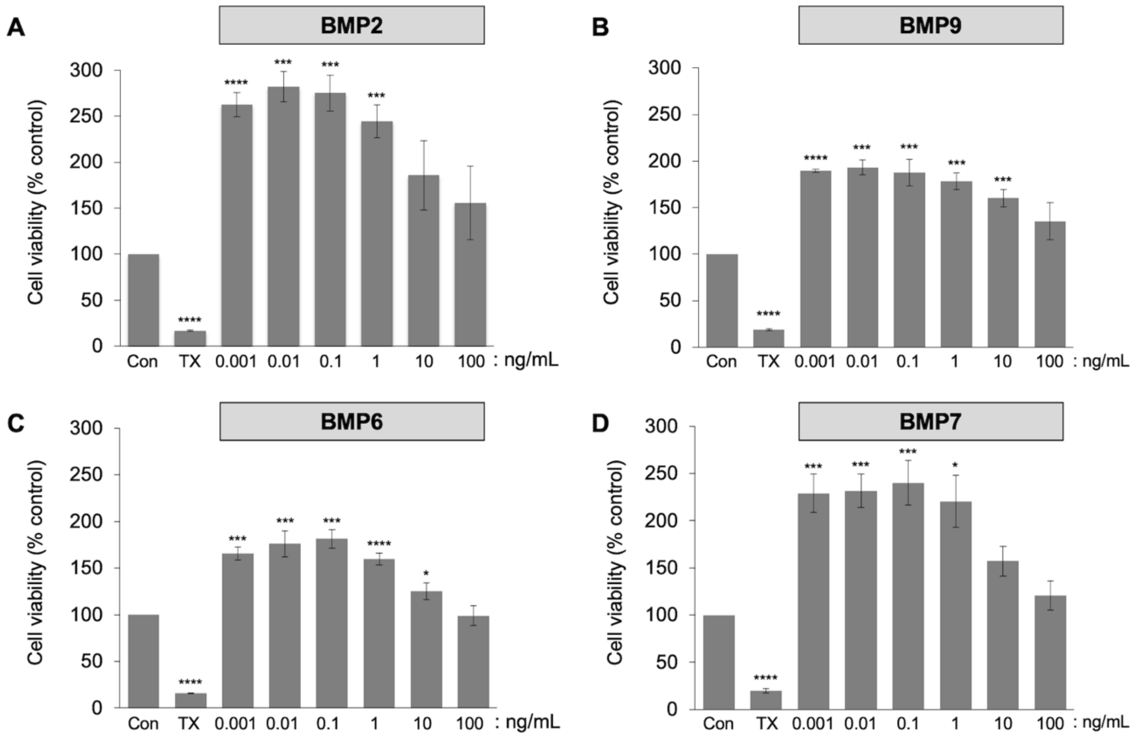

Supplemental Figure S3. BMPs increases the cell viability in C2C12 cells. Serum-starved C2C12 cells were treated with the indicated concentrations of (A) BMP2, (B) BMP9, (C) BMP6 and (D) BMP7 for 24 hours. Treatments with Triton X-100 (TX, 125 µM) was used as negative control. Cell viability is presented as a percent of luminescence detected in control, untreated cells (mean ± SEM (n=3)). BMPs representing three distinct BMP sub-families enhanced cell viability after 24 hours of treatment at very low concentrations compared to the control, untreated C2C12 cells. BMP2: (0.001 µg/mL, 263%, ****$p < 0.0005$; 0.01 µg/mL, 282%, 0.1 µg/mL, 275%, 1 µg/mL, 245%, ***$p < 0.001$; 10 µg/mL, 186%; 100 µg/mL, 156%, not significant); BMP9: (0.001 µg/mL, 190%, ****$p < 0.0005$; 0.01 µg/mL, 193%, 0.1 µg/mL, 188%, 1 µg/mL, 178%, 10 µg/mL, 160%, ***$p < 0.001$; 100 µg/mL, 135%, not significant);

BMP6: (0.001 μg/mL, 165%, 0.01 μg/mL, 176%, 0.1 μg/mL, 181%, ***$p < 0.001$; 1 μg/mL, 160%, ****$p < 0.0005$; 10 μg/mL, 125%, *$p < 0.05$; 100 μg/mL, 99%, not significant); BMP7: (0.001 μg/mL, 229%, 0.01 μg/mL, 232%, 0.1 μg/mL, 240%, ***$p < 0.001$; 1 μg/mL, 220%, *$p < 0.05$; 10 μg/mL, 157%, 100 μg/mL, 121%, not significant).

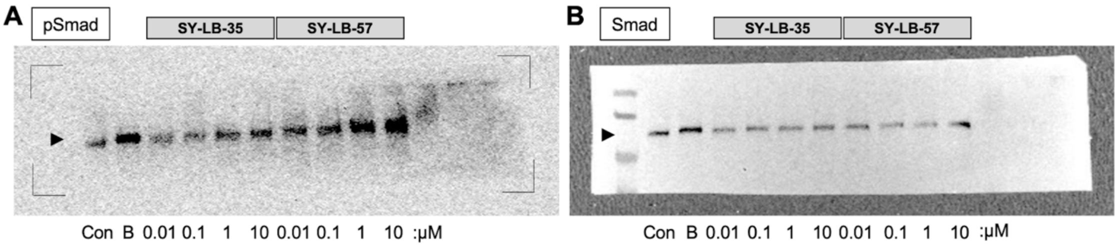

Supplemental Figure 4. p-Smad and total Smad Western blot images from Figure 5. The full, uncropped nitrocellulose membranes incubated with (A) anti-p-Smad and (B) anti-Smad1 antibodies. The p-Smad blot was stripped and re-probed for Smad. The image in (B) is a merge of the Marker image and the exposure shown. Brackets in (A) indicate edges of nitrocellulose membrane shown in (B). Arrowheads indicate the (A) p-Smad and (B) Smad bands at 52 kDa. The marker bands visible in (B) in descending size are 100, 75 and 50 kDa.

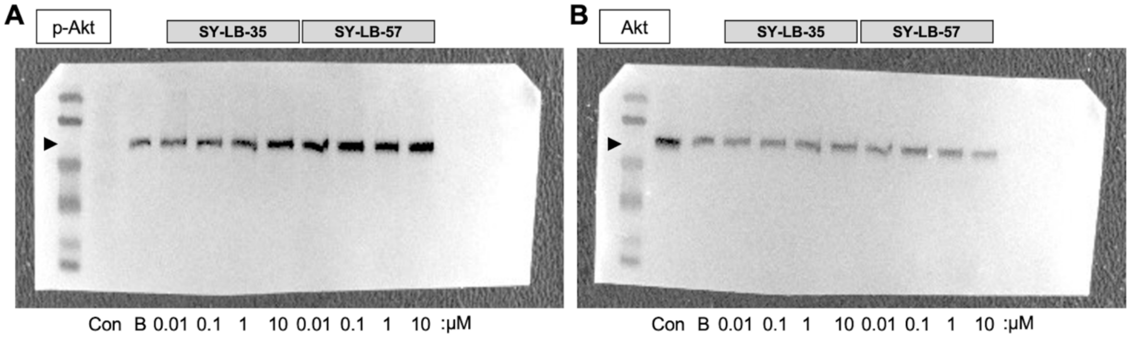

Supplemental Figure S5. p-Akt and total Akt Western blot images from Figure 6. The full, uncropped nitrocellulose membranes incubated with (A) anti-p-Akt and (B) anti-total Akt antibodies. The p-Akt blot was stripped and re-probed for Akt. Arrowheads indicate the (A) p-Akt and (B) Akt bands at 62 kDa. The marker bands visible in (A) and (B) in descending size are 100, 75, 50, 37, 25 and 20 kDa.

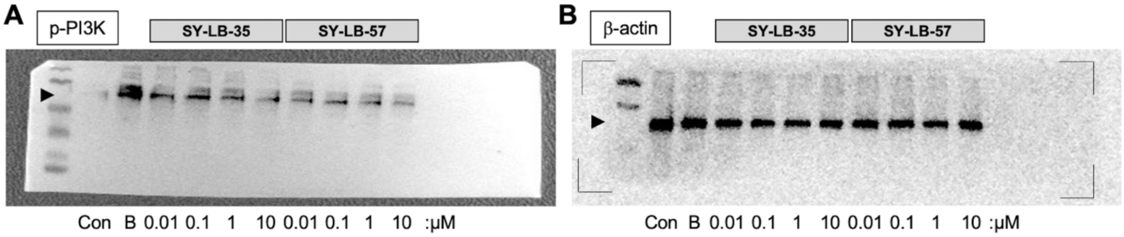

Supplemental Figure S6. p-PI3K and β-actin Western blot images from Figure 7. The full, uncropped nitrocellulose membranes incubated with (A) anti-PI3K and (B) anti-β-actin antibodies. The p-PI3K blot was stripped and re-probed for β-actin. The image in (A) is a merge of the Marker image and the exposure shown. Brackets in (B) indicate edges of nitrocellulose membrane shown in (A). Arrowheads indicate the (A) p-PI3K bands at 55 kDa and (B) β-actin bands at 42 kDa. The marker bands visible in (A) in descending size are 100, 75, 50, 37, 25 and 20 kDa.

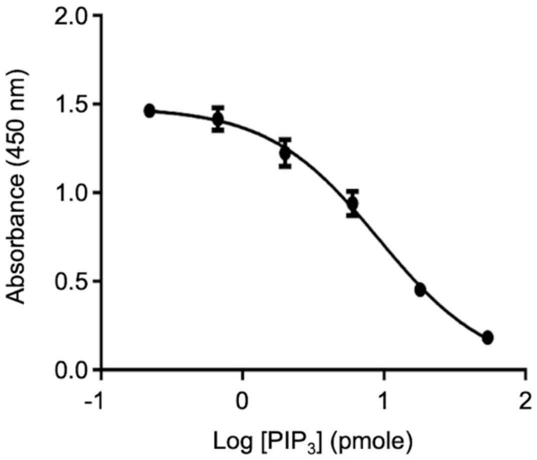

Supplemental Figure S7. PIP_3 standard curve. A standard curve of the log of PIP_3 concentrations of (0.22, 0.67, 2, 6, 18, 54 picomolar (pmole)) versus absorbance at 450 nm was plotted. The amount of PIP_3 produced in each treatment sample was calculated by interpolation from the standard curve.

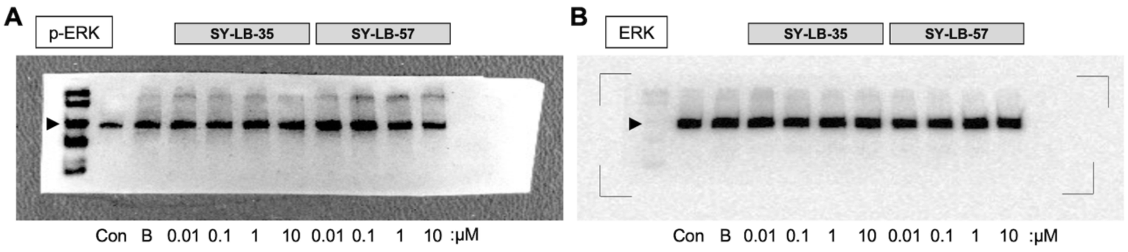

Supplemental Figure S8. p-ERK and ERK Western blot images from Figure 8. The full, uncropped nitrocellulose membranes incubated with (A) anti-p-ERK and (B) anti-total ERK antibodies. The p-ERK blot was stripped and re-probed for ERK. The image in (A) is a merge of the Marker image and the exposure shown. Brackets in (B) indicate edges of nitrocellulose membrane shown in (A). Arrowheads indicate the (A) p-ERK and (B) ERK bands at 42 kDa. The marker bands visible in (A) in descending size are 100, 75, 50, 37, 25 and 20 kDa.

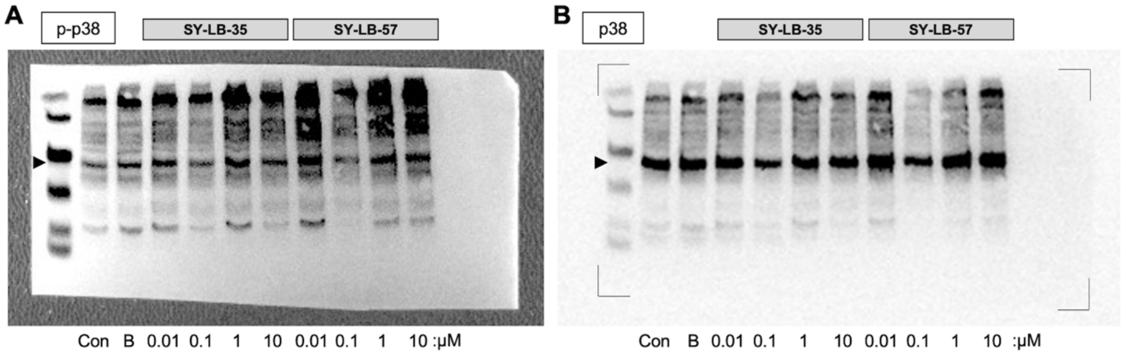

Supplemental Figure S9. p-p38 and p38 Western blot images from Figure 8. The full, uncropped nitrocellulose membranes incubated with (A) anti-p-p38 and (B) anti-p38 antibodies. The p-p38 blot was stripped and re-probed for p38. The image in (A) is a merge of the Marker image and the exposure shown. Brackets in (B) indicate edges of nitrocellulose membrane shown in (A). Arrowheads indicate the (A) p-p38 and (B) p38 bands at 41 kDa. The marker bands visible in (A) and (B) in descending size are 100, 75, 50, 37, 25 and 20 kDa.

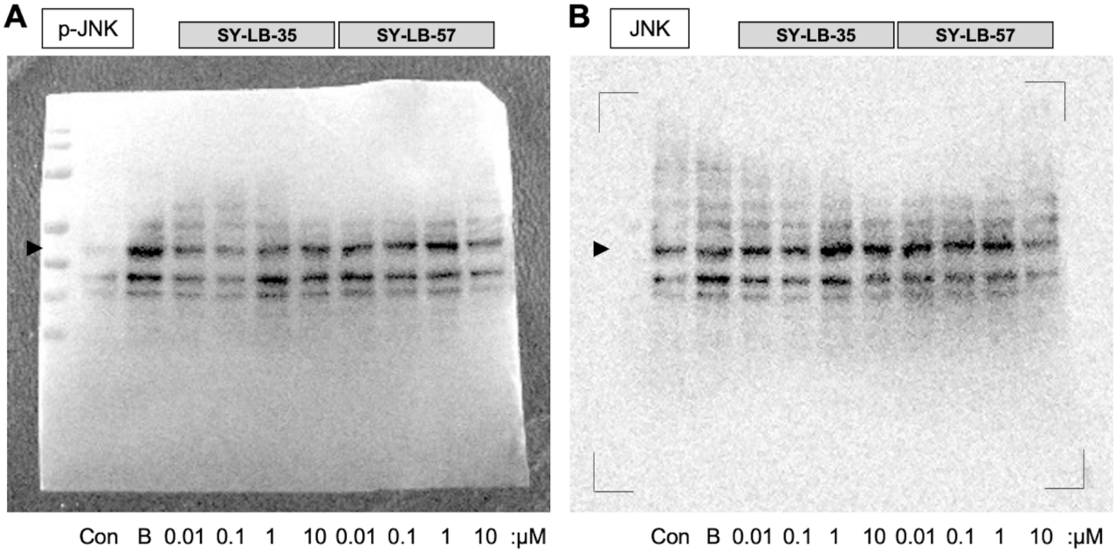

Supplemental Figure S10. p-JNK and JNK Western blot images from Figure 8. The full, uncropped nitrocellulose membranes incubated with (A) anti-p-JNK and (B) anti-JNK antibodies. The p-JNK blot was stripped and re-probed for JNK. The image in (A) is a merge of the Marker image and the exposure shown. Brackets in (B) indicate edges of nitrocellulose membrane shown in (A). Arrowheads indicate the (A) p-JNK and (B) JNK bands at 48 kDa. The marker bands visible in (A) in descending size are 100, 75, 50, 37, 25 and 20 kDa.

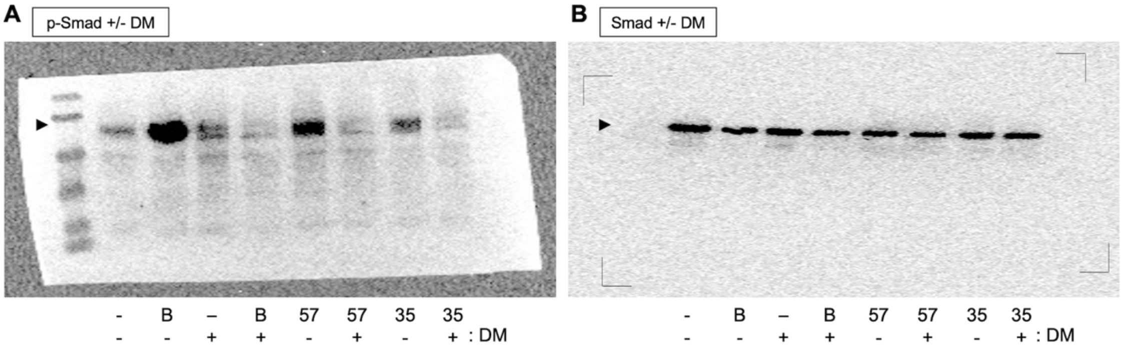

Supplemental Figure S11. p-Smad and Smad Western blot images from Figure 10. The full, uncropped nitrocellulose membranes incubated with (A) anti-p-Smad and (B) anti-Smad1 antibodies. The p-Smad blot was stripped and re-probed for Smad. The image in (A) is a merge of the Marker image and the exposure shown. Brackets in (B) indicate edges of nitrocellulose membrane shown in (A). Arrowheads indicate the (A) p-Smad and (B) Smad bands at 52 kDa. The marker bands visible in (A) in descending size are 100, 75, 50, 37, 25 and 20 kDa.
